# Muskox status, recent variation, and uncertain future

**DOI:** 10.1007/s13280-019-01205-x

**Published:** 2019-06-11

**Authors:** Christine Cuyler, Janice Rowell, Jan Adamczewski, Morgan Anderson, John Blake, Tord Bretten, Vincent Brodeur, Mitch Campbell, Sylvia L. Checkley, H. Dean Cluff, Steeve D. Côté, Tracy Davison, Mathieu Dumond, Barrie Ford, Alexander Gruzdev, Anne Gunn, Patrick Jones, Susan Kutz, Lisa-Marie Leclerc, Conor Mallory, Fabien Mavrot, Jesper Bruun Mosbacher, Innokentiy Mikhailovich Okhlopkov, Patricia Reynolds, Niels Martin Schmidt, Taras Sipko, Mike Suitor, Matilde Tomaselli, Bjørnar Ytrehus

**Affiliations:** 1 Greenland Institute of Natural Resources, PO Box 570, 3900 Nuuk, Greenland; 2 School of Natural Resources and Extension, University of Alaska Fairbanks, Fairbanks, AK 99775 USA; 3 Wildlife Division, Environment and Natural Resources, Government of Northwest Territories, PO Box 1320, Yellowknife, NT X1A 2L9 Canada; 4 BC Ministry of Forests, Lands, Natural Resources Operations and Rural Development, 2000 South Ospika Blvd, Prince George, BC V2N 4W5 Canada; 5 Animal Resources Center, University of Alaska Fairbanks, PO Box 756980, Fairbanks, AK 99775 USA; 6 Norwegian Environment Agency, PO Box 5672 Torgarden, 7485 Trondheim, Norway; 7 Department of Wildlife Management of Northern Québec, Ministry of Forests, Wildlife and Parks of Québec, 951 Hamel Boulevard, Chibougamau, QC G8P 2Z3 Canada; 8 Department of Environment, Government of Nunavut, PO Box 120, Arviat, NT X0C 0E0 Canada; 9 Department of Ecosystem and Public Health, Faculty of Veterinary Medicine, University of Calgary, 3280 Hospital Drive NW, Calgary, AB T2N 4Z6 Canada; 10 Environment and Natural Resources, Government of the Northwest Territories, PO Box 2668, 3803 Bretzlaff Drive, Yellowknife, NT X1A 2P9 Canada; 11 Département de biologie & Centre for Northern Studies, Université Laval, 1045 avenue de la Médecine, Québec, G1V 0A6 Canada; 12 Department of Environment and Natural Resources, Wildlife Management, Inuvik Region, PO Box 2749, Inuvik, NT X0E 0T0 Canada; 13 Umingmak Productions Inc., Kugluktuk, NU X0B 0A2 Canada; 14 Nunavik Research Centre, Makivik Corporation, PO Box 179, Kuujjuaq, QC J0M 1C0 Canada; 15 Wrangel Island State Reserve, Pevek, Russia 689400; 16 368 Roland Road, Salt Spring Island, V8K 1V1 BC Canada; 17 Division of Wildlife Conservation, Alaska Department of Fish and Game, PO Box 1467, Bethel, AK 99559 USA; 18 Department of Environment, Government of Nunavut, PO Box 377, Kugluktuk, NU X0B 0A2 Canada; 19 Department of Environment, Government of Nunavut, PO Box 209, Iglulik, NU X0A 0L0 Canada; 20 Institute of Biological Problems of Cryolithozone of the Siberian Branch of Russian Academy of Science (IBPC SB RAS), 41 Lenina Ave., Yakutsk, Russia 677980; 21 PO Box 80843, Fairbanks, AK 99775 USA; 22 Arctic Research Centre, Department of Bioscience, Aarhus University, Frederiksborgvej 399, 4000 Roskilde, Denmark; 23 Severtsov Institute of Ecology and Evolution, Russian Academy of Sciences, PO Box 11, Moscow, Russia 119071; 24 Inuvialuit and Migratory Caribou, Fish and Wildlife, Environment Yukon, PO Box 600, Dawson City, YT Y0B 1G0 Canada; 25 Polar Knowledge Canada, Canadian High Arctic Research Station, 1 Uvajuq Road, PO Box 2150, Cambridge Bay, NU X0B 0C0 Canada; 26 Norwegian Institute for Nature Research (NINA), PO Box 5685 Torgarden, 7485 Trondheim, Norway

**Keywords:** Abundance, Circumpolar, Drivers, *Ovibos*, Population status, Trends

## Abstract

Muskoxen (*Ovibos moschatus*) are an integral component of Arctic biodiversity. Given low genetic diversity, their ability to respond to future and rapid Arctic change is unknown, although paleontological history demonstrates adaptability within limits. We discuss status and limitations of current monitoring, and summarize circumpolar status and recent variations, delineating all 55 endemic or translocated populations. Acknowledging uncertainties, global abundance is ca 170 000 muskoxen. Not all populations are thriving. Six populations are in decline, and as recently as the turn of the century, one of these was the largest population in the world, equaling ca 41% of today’s total abundance. Climate, diseases, and anthropogenic changes are likely the principal drivers of muskox population change and result in multiple stressors that vary temporally and spatially. Impacts to muskoxen are precipitated by habitat loss/degradation, altered vegetation and species associations, pollution, and harvest. Which elements are relevant for a specific population will vary, as will their cumulative interactions. Our summaries highlight the importance of harmonizing existing data, intensifying long-term monitoring efforts including demographics and health assessments, standardizing and implementing monitoring protocols, and increasing stakeholder engagement/contributions.

**Electronic supplementary material:**

The online version of this article (10.1007/s13280-019-01205-x) contains supplementary material, which is available to authorized users.

## Introduction

**Fig. 1:**
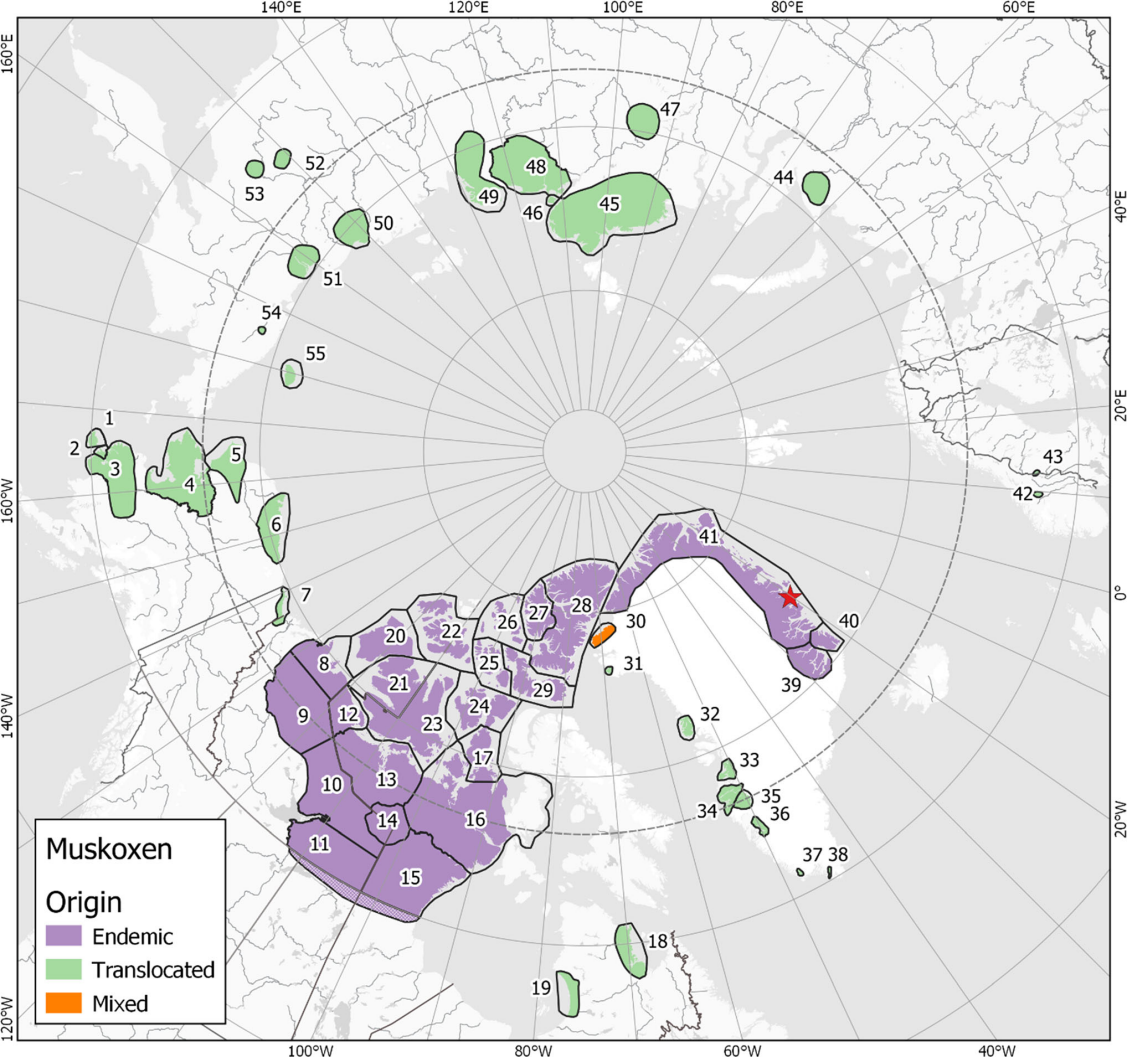
Global overview of current distribution and origin of muskox populations: endemic, translocated, and mixed. Translocated includes introduced and re-introduced, i.e., to range once occupied either in recent or distant past. Mixed is translocation to an area with endemic muskoxen. Numbering corresponds with Table 1, and indicates an administrative region, a management unit, or a population. The provided boundaries are guidelines, often reflecting administrative or political regions. They are not a precise distribution/extent for a specific population, e.g., since muskoxen can and do travel across sea-ice, even the islands are not strict boundaries. The muskox distribution in central Canada around 60°N is uncertain owing to anecdotal observations and low animal density. Populations 3, 7, 19, 34, and 36 originated as range expansions by translocated populations. Zackenberg Station is the red star in NE Greenland (see Electronic Supplementary Materials S1, Muskoxen: Past and present). Dashed line is the Arctic Circle

For the past 50 years, the Arctic has been warming twice as fast as the rest of the world creating a climate that today is warmer, wetter, and increasingly more variable (AMAP 2017). Apprehension about the impact of changing climate on Arctic ecosystems is growing in the face of many unknowns. This paper focuses on the muskox (*Ovibos moschatus*), a large-bodied herbivore that plays a central role in many Arctic ecosystems. It is physiologically and behaviorally adapted to living year-round in the Arctic. Today, muskox populations (endemic and translocated/re-introduced) inhabit a range that extends from sub- to high Arctic (56°–83°N) environments (Fig. 1).

Muskoxen have an intrinsic connection with the culture, traditions, and heritage of Arctic indigenous peoples, a connection that continues to evolve (Tomaselli et al. 2018a). They are an important food resource in an area of increasing food insecurity and they provide diverse economic opportunities where few exist (Kutz et al. 2017).

Two subspecies, *O.m. wardi* and *O.m. moschatus*, are commonly recognized and referred to as ‘White-Faced’ and ‘Barren-Ground,’ respectively (van Coeverden de Groot 2001), and recent studies have identified genetic separation between the two (Hansen et al. 2018). We therefore refer to the two subspecies throughout this study.

In 2014, the Muskox Expert Network (MOXNET) emerged from the mammalian component of the terrestrial Circumpolar Biodiversity Monitoring Program (CBMP). Participants from seven circumpolar countries, representing government and non-governmental agencies, indigenous peoples, businesses, and academics, came together to establish a network of experts for the sharing and exchange of information on muskoxen. This paper is a MOXNET collaborative compilation of the current information on muskoxen. Following the protocols outlined in the Arctic Terrestrial Biodiversity Monitoring Plan (CBMP Terrestrial Steering Group 2015), we present estimates and information on muskox population abundance and distribution, and discuss demographics, spatial distribution, health, and genetic diversity. Within this context, we identify primary drivers of change and stressors potentially influencing muskox population dynamics along with important knowledge gaps. Finally, we summarize key findings and suggest recommendations in an effort to foster sustainable muskox populations throughout the circumpolar north during a changing and uncertain future.

## Methods

We updated the global distribution and origins of muskox populations reported in Kutz et al. (2017) and added current population/region boundaries. The boundaries provided often reflect administrative or political regions rather than specific muskox populations and their actual distribution within a region. Therefore, these boundaries do not necessarily reflect population structures, and are likely to change as protocols for standardizing biologically meaningful population boundaries are established and implemented.

We compiled current abundance estimates for the 55 geographic regions with muskox populations (Table 1). These estimates include all age classes. The majority (80%) of our population sizes are based on surveys within the past decade. Further, over half of these were monitored recently, i.e., in the period 2016–2019 (54.5%; *n* = 30) and 25.5% (*n* = 14) within 2009–2015. Where geographic regions surveyed subareas piecemeal, a sum total estimate was provided for the region. Electronic Supplementary Materials contain details on recent and past abundance estimates for each population (Excel Table S3).

**Table 1:** Global overview of muskox populations, location, subspecies designation, CAFF Arctic zone (CAFF 2013), last survey year, population size, and recent variation (suggested trend) within the last 10 years (Electronic Supplementary Materials, Excel Table S3 contains details)

Country/Muskox population	Figure 1 no.	Subspecies	CAFF Arctic zone	Last survey year	Population size^a^	Recent variation
**USA—Alaska**						
Nunivak Island	1	*wardi*	Low	2015	740	Stable
Nelson Island	2	*wardi*	Low	2018	444	Stable
Yukon Kuskokwim Delta	3	*wardi*	Low	2017	252	Increasing^b^
Seward Peninsula	4	*wardi*	Low	2017	2353	Stable
Cape Thompson	5	*wardi*	Low	2017	227	Decreasing
North East	6	*wardi*	Low	2018	285	Increasing
***Total Alaska***					**ca 4301**	
**Canada Mainland**						
*Yukon*						
Yukon North slope	7	*wardi*	Low	2018	344	Increasing
*Northwest Territories*						
Inuvik	8	*moschatus*	Low/sub	2009	2855	Stable
Sahtu	9	*moschatus*	Sub	1997	1457	Increasing
North Great Slave	10	*moschatus*	Sub	2018	8098	Increasing
South Great Slave	11	*moschatus*	Sub	2011	164	Increasing^c^
*Nunavut*						
MX-09	12	*moschatus*	Low	2018	539	Stable
MX-11^d^	13	*moschatus*	Low	2013	13 592	Unknown
Thelon, MX-12	14	*moschatus*	Low/sub	1994	1095	Decreasing
MX-13	15	*moschatus*	Low/sub	2010	4736	Increasing
MX-10^e^	16	*moschatus*	High/low	2013	3685	Increasing
Boothia Peninsula MX-08	17	*wardi*	High	2018	3649	Increasing
*Quebec (Nunavik)*						
Ungava Bay	18	*wardi*	Low	2019	3000	Increasing
Eastern Hudson Bay	19	*wardi*	Low/sub	2016	1000	Increasing
**Canada Arctic Archipelago**^f^						
*Northwest Territories*						
Banks Is.	20	*wardi*	High	2014	14 021	Decreasing
NW. Victoria Is.	21	*wardi*	High	2015	14 547	Stable
Melville Is. Complex^g^	22	*wardi*	High	2012	3716	Increasing
* Nunavut*						
E. Victoria Is. MX-07	23	*wardi*	High	2014	10 026	Decreasing
Pr. Wales/Somerset Is.^g^ MX-06	24	*wardi*	High	2016	3052	Unknown
Bathurst Is. Complex^g^ MX-05	25	*wardi*	High	2013	1888	Increasing
Ringnes & Cornwall Is. MX-03	26	*wardi*	High	2007	21	Unknown
Axel Heiberg Is. MX-02	27	*wardi*	High	2007	4237	Unknown
Ellesmere Is. MX-01	28	*wardi*	High	2015	11 315	Increasing
Devon Is. MX-04	29	*wardi*	High	2016	1963	Increasing
***Total Canada***					**ca 109 000**	
**Greenland**						
Inglefield Land	30	*wardi*	High	2000	273	Unknown
Cape Atholl	31	*wardi*	High	2017	212	Stable
Sigguk (Svartenhuk)	32	*wardi*	Low	2002	193	Unknown
Naternaq	33	*wardi*	Low	2004	112	Unknown
Sisimiut	34	*wardi*	Low	2018	2622	Unknown
Kangerlussuaq	35	*wardi*	Low	2018	20 334	Unknown
Nuuk	36	*wardi*	Low	2016	14	Unknown
Ivittuut	37	*wardi*	Low	2017	812	Decreasing^h^
Nanortalik	38	*wardi*	Sub	2018	32	Increasing
Inner Kangertittivaq Fjord	39	*wardi*	High	2004	562	Unknown
Jameson Land	40	*wardi*	High	2000	1761	Unknown
North East Greenland	41	*wardi*	High	1992	12 500	Unknown
***Total Greenland***					**ca 39 427**	
**Scandinavia**						
Norway: Dovre	42	*wardi*	Not Arctic	2018	244	Stable
Sweden: Rogen Nature Reserve	43	*wardi*	Not Arctic	2017	10	Unknown
*** Total Scandinavia***					**ca 254**	
**Russia**						
Yamal Peninsula^i^	44	*wardi*	Low	2017	300	Increasing
Taimyr Peninsula	45	*wardi*	Low	2017	12 100	Increasing
Begicheva Island^j^	46	*wardi*	Low	2017	230	Stable
Putorana Plateau	47	*wardi*	Sub	2004	20	Unknown
Anabarskay	48	*wardi*	Low/sub	2017	1040	Increasing
Bulunskay^k^	49	*wardi*	Low/sub	2017	700	Increasing
Indigirskay	50	*wardi*	Low/sub	2017	350	Increasing
Kolymskay	51	*wardi*	Low/sub	2017	30	Increasing
Magadan Oblast	52	*wardi*	Sub	2015	16	Unknown
Magadan Omulevka River	53	*wardi*	Sub	2015	6	Unknown
Chukotka^l^	54	*wardi*	Low	2017	4	Decreasing
Wrangel Island	55	*wardi*	Low	2018	1000	Increasing
*** Total Russia***					**ca 15 796**	
**GLOBAL TOTAL MUSKOXEN**					**ca 168 778**	

^a^Size indicates a recent estimate or a minimum/total count (see Electronic Supplementary Materials, Excel Table S3)

^b^Local knowledge and observations indicate increasing abundance and distribution

^c^Recent variation is for 2018; based on increasing number of opportunistic sightings, possibly stabilizing by 2018

^d^Currently includes Kugluktuk, Queen Maud, Contwoyto Lake, and two old regions: MX-14 and MX-19. Kuglugtuk sub-area, last surveyed in 2013, may be increasing

^e^Currently includes King William Is, Adelaide Peninsula, and two old regions: MX-17 and MX-20

^f^Only major island names provided

^g^Melville Is. complex, includes Melville, Prince Patrick, and Eglinton Islands. Bathurst Is. complex includes Bathurst, Cornwallis, Little Cornwallis, Helena, Sherard-Osborn, Cameron, Vanier, Massey, and Alexander Islands. Prince of Wales/Somerset Island also includes Russell, Prescott, and Pandora Islands

^h^Harvest management induced decline

^i^2016, An additional 60 muskoxen were translocated from the Aviary (captive breeding facility)

^j^2017-Survey method permitted more accurate count than previously, thus not assumed an increase in herd size

^k^2017, An additional 22 muskoxen translocated to the Lena River Delta

^1^Although muskoxen have been released several times (most recently in 2010), bears/humans cause high mortality

**Fig. 2:**
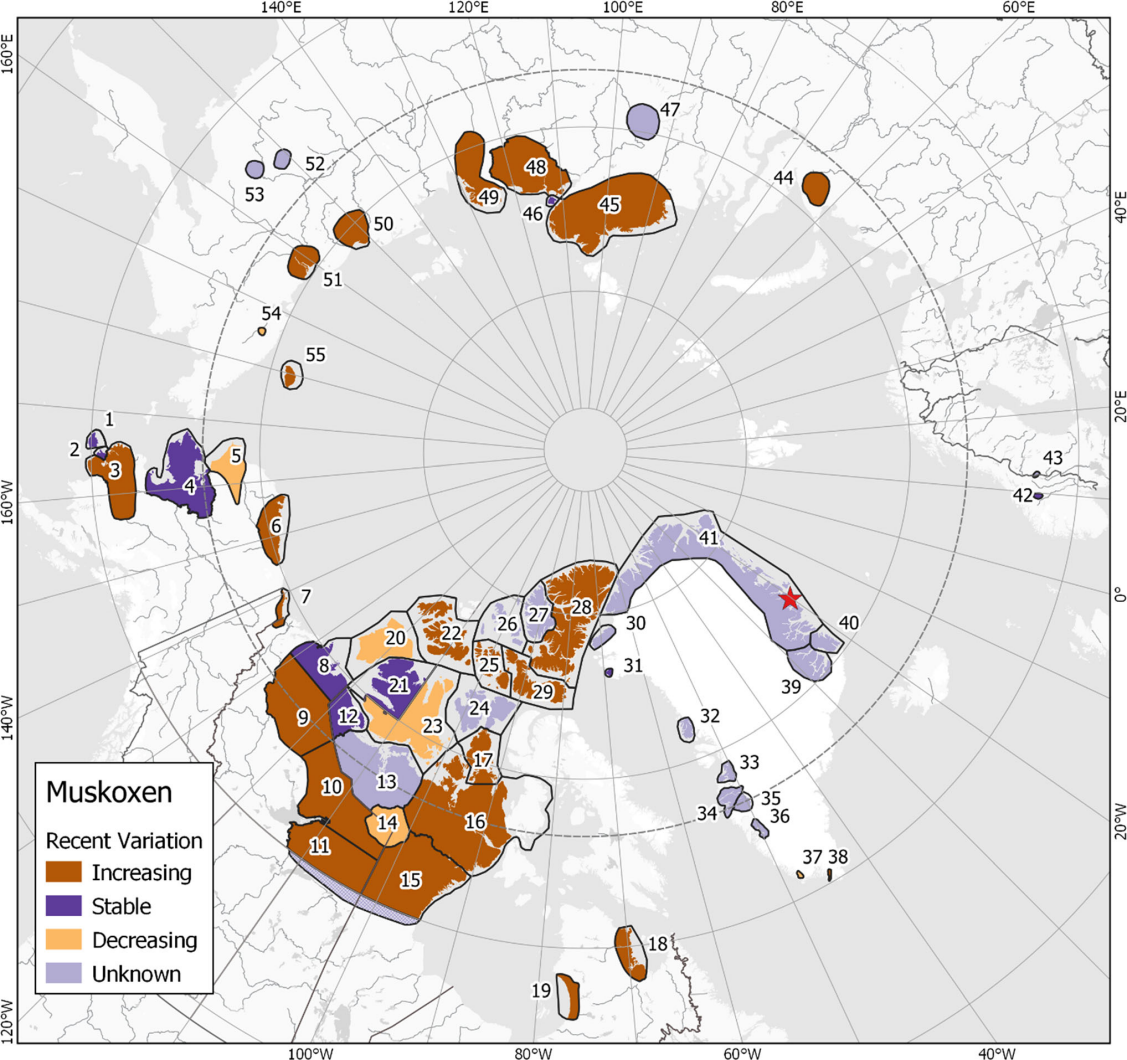
Global overview of recent variation in muskox abundance. Numbering corresponds with Table 1 and indicates an administrative region or population. The provided boundaries are guidelines and not precise distributions of a given population. Zackenberg Station is the red star in NE Greenland (see Electronic Supplementary Materials S1, Muskoxen: Past and present). Dashed line is the Arctic Circle

Statistical trend analyses for abundance of a specific population were rarely possible, because surveys were often too infrequent, had unavailable estimates of variance, or had different methods or effort between surveys. Thus, we provide the most recent abundance estimate (Table 1), and used abundance changes over the last 10 years (Electronic Supplementary Materials, Excel Table S3) to reveal recent variation, suggesting possible trends (Fig. 2). Estimates, counts, and recent variation were corroborated by local experts (regional biologists, research scientists) wherever possible (Electronic Supplementary Materials, Muskoxen: Past and present, and Excel Table S3). Recent variation/trend was labeled unknown if the estimate/count was older than 10 years, a recent once-only effort, or involved ≤ 20 individuals and additional expert knowledge was unavailable.

## Results and discussion

Of all the Focal Ecosystem Component (FEC) attributes prioritized for terrestrial mammals in the Arctic Terrestrial Biodiversity Monitoring Plan (Christensen et al. 2013), estimates of muskox abundance comprise the most extensive data available both geographically and temporally. Despite the limitations and inconsistencies in the data, our best approximation of current global abundance is 170 000 muskoxen, of which 71% are endemic (Table 1). While some populations are in decline (e.g., Banks and Victoria islands), others have expanded their range or experienced increases typical of translocated populations (see Electronic Supplementary Materials, Muskoxen: Past and present S1, and Excel Table S3). Occasionally, a stable or decreasing population trend is the result of wildlife management interventions designed around specific goals (e.g., Nunivak Island and Ivittuut respectively, see Electronic Supplementary Materials S1, Muskoxen: Past and present). Translocations over the past century have resulted in a circumpolar distribution of muskoxen, and all re-introduced/translocated animals have been *O.m. wardi* (see Electronic Supplementary Materials, Excel Table S3). The combined number of re-introduced, translocated, and endemic *O.m. wardi* (e.g., 132 557) now vastly outnumber *O.m. moschatus* (e.g., 36 221), which remain confined primarily to mainland Canada. Nevertheless, endemic muskoxen (both *O.m. wardi* and *O.m. moschatus*) still outnumber re-introduced/translocated muskoxen, e.g., 119 479 to 49 026, respectively (the mixed population of Inglefield Land not included). Given already low genetic variability among endemic sources (Groves 1997; Holm et al. 1999) and the relatively few individuals captured for translocations (often from the same geographic source), future studies may reveal exacerbated low variability in several translocated populations. More information on successful and failed translocations is available in Electronic Supplementary Materials (S1 Muskoxen: Past and present).

Our circumpolar estimate of 170 000 is greater than previous estimates of 134 000–137 000 (IUCN 2008), ca 135 000 (Gunn et al. 2013), and 111 000–135 000 (Kutz et al. 2017), and represents our best approximation given all data ambiguities. The compiled abundance surveys commonly gave estimates that contained all age classes. Thus, we were unable to provide a circumpolar estimate of only reproductive adults, although this is the criterion implemented by IUCN.

We could suggest recent trends for 38 out of our 55 muskox populations/regions based on variation over the past decade (Fig. 2). Of these, 23 appear to be increasing. These represent 36.2% (*n* = 61 104) of present global abundance. Similarly, nine populations appear stable and six decreasing, representing 13.1% (*n* = 22 164) and 15.5% (*n* = 26 185), respectively, of present global abundance. It is worth noting that two of the declining populations were once the largest endemic populations in the world, i.e., Banks and East Victoria islands in Canada. At the turn of the century, these two combined totaled ca 87 000 muskoxen, but today they are ca 24 000 (see Electronic Supplementary Materials, Excel Table S3). Mortality events caused by infectious agents have been identified in both regions (see Electronic Supplementary Materials S1, Muskoxen: Past and present). The fact that recent trends are unknown for a further 17 populations (35.1%; *n* = 59 322) makes it difficult to interpret the true impact of these declines relative to the total global population. Regardless, it is clear that population status can change quickly.

### Abundance

We recognize that natural fluctuations in population size are normal, often unpredictable, and not always synonymous with long-term trends, and thus abundance data and suggested trends are not without their limitations. Regardless, they provide some context where previously little existed. Muskox ranges are remote and cover vast areas, often crossing jurisdictional boundaries. Few are near human settlements or airports, making aerial surveys expensive and logistically difficult. Sample counts using line or strip transects are commonly used to estimate muskox abundance. However, area coverage varies and so does precision. For example, the coefficient of variation (CV) for 17 estimates on Banks Island (Canada) averaged 11% but was 30% for two surveys on the mainland (Queen Maud Gulf coast, Canada). Additionally, detection (sightability) of muskoxen present on a survey line varies. Detection is affected by distance from survey line, group size, terrain features determining viewing distance, weather conditions, and type of background (e.g., variations in the ratio of snow cover to bare ground/boulders/vegetation poking through snow surface), as well as animal movements or lack thereof. Observer ability, fatigue, and airsickness also influence the detection of animals present on a survey line. Poor sightability can underestimate population abundance.

Assessment of trends in muskox abundance over time and across regions is complicated further by variable survey methods and inconsistent survey efforts (extent of area covered) within the same region. The recent change to Nunavut’s muskox management units/regions exacerbates existing obstacles to making trend assessments. Among study areas, different survey methods are often employed. For example, Nelson Island, AK, is a relatively small survey area. Here, by using small aircraft and employing photography with close line spacing, surveys produce results that approximate a total count (Jones 2015). On Banks Island, strip-transect fixed-wing surveys with consistent methods and coverage have been used since the 1980s (Davison et al. 2017). However, due to changes in terrain across the Canadian High Arctic, surveys of muskoxen in Nunavut have employed both helicopter-based distance-sampling methods (Jenkins et al. 2011) and fixed-wing strip-transect methods (Anderson and Kingsley 2017). A complex terrain and financial constraints challenge Greenland surveys. Unsystematic ground counts have been typical, although there have been some fixed-wing or helicopter strip counts, and recently, the Sisimiut and Kangerlussuaq populations were assessed using helicopter-based distance sampling. Regardless, with the exception of Zackenberg and Ivittuut, Greenland surveys are infrequent or provide a one-time snapshot for now. While a more consistent approach on a large scale is desirable for surveys of muskoxen, local and regional conditions and topography, together with limitations of funds and staff, mean that the mosaic of survey methods is likely to continue. Recognizing these difficulties, the goal remains a standardization of field methods, the absence of which makes rigorous statistical trend analyses impossible. We must establish and implement protocols for defining what constitutes a muskox population, thus forming the basis for consistent, uniformly defined survey areas. We also require standardized monitoring protocols, among these, how to incorporate the traditional and local knowledge that can supplement infrequent surveys. Once standards for the above gain broader acceptance and implementation, comparing trends across regions can be done with statistical confidence and certainty.

### Demographics

Annual recruitment affects future population trend (Schmidt et al. 2015), regardless of present abundance. The ultimate influence of drivers and stressors on muskox populations is how these affect vital rates for calf births, calf survival, and adult survival. These three rates are integral to population trends. Knowledge about muskox demographics is however hard to obtain, as demographic monitoring is not widespread and published data are scarce. The necessary ground-based surveys, ideally incorporating the use of telemetry (collared animals), are logistically difficult and usually expensive. Studies to date involve only small populations, or areas of high density. Additionally, group composition varies depending on season (Schmidt et al. 2015), which confounds comparison of sex and age structure surveys. The natural mortality rate for adults, although unknown, may be approximated for a specific population if average life expectancy is available.

Monitoring demographics is among the protocols outlined in the Arctic Terrestrial Biodiversity Monitoring Plan (Christensen et al. 2013). We recognize that reliable demographic information is vital for developing relevant management strategies and policy. Consistent, standardized approaches for gathering seasonal demographics are essential for accurately interpreting abundance trends and will enhance our ability to compare population dynamics across regions.

### Spatial distribution and genetic diversity

Although generally not considered migratory, seasonal distributions of muskoxen can span broad geographic regions (Fig. 1). To take advantage of forage quality and accessibility, groups may move between winter and summer ranges (Tener 1960; Gunn and Fournier 2000), while in other areas habitat heterogeneity allows muskoxen a more sedentary lifestyle (Schmidt et al. 2016). Further, striking shifts in range use have also been observed, with muskoxen in northeastern Alaska having expanded their range into adjacent regions and vacating originally occupied areas (Reynolds 2011). Mixed groups will occasionally leave to colonize an entirely different region (Cuyler pers. comm.), even moving across glacial barriers (Schmidt et al. 2016). The wide dispersion of this species and these relatively unpredictable movements impede survey efforts, especially when coupled with infrequent surveys (Adamczewski in Kutz et al. 2017).

Muskoxen are among a handful of Arctic species that survived major shifts in climate (Raghavan et al. 2014). The archeological record, supported by genetic data (MacPhee et al. 2005), provides evidence that muskoxen have been through several population bottlenecks and extirpation events that are best explained by non-anthropogenic causes, e.g., environmental change (Campos et al. 2010). This has left present day muskoxen challenged by low genetic variability (Hansen et al. 2018) and extremely low diversity in the major histocompatibility complex, potentially impacting their ability to respond to infectious disease (Gordeeva et al. 2009; Cooley et al. 2011; Thulin et al. 2011). A better understanding of muskox genetics would be instrumental in steering future management and conservation efforts.

### Health

Although the need for monitoring disease in muskoxen was recognized almost 80 years ago (Jennov 1941), attention to muskox diseases is relatively new with only sporadic accounts of infectious diseases and parasites in the early literature (Tener 1965; Mathiesen et al. 1985). Recent documentation has occurred in connection with declining populations where emerging pathogens and shifting disease dynamics have been observed. For example, acute and extensive infectious disease associated summer mortalities in Alaska and Canada coincided with population declines of up to 85% (Kutz et al. 2015; Forde et al. 2016), and outbreaks of *Pasteurella* spp., *Mycoplasma* spp., and parapox virus in muskoxen in the Dovrefjell, Norway, have been identified in declining populations (Ytrehus et al. 2008, 2015; Handeland et al. 2014). Changing pathogen distribution and disease dynamics have also been observed with climate-driven range expansion of the lung nematode *Umingmakstrongylus pallikuukensis* in the Northwest Territories and Nunavut (Kutz et al. 2013a, b; Kafle et al. 2017), the emergence of parapox virus, and increasing observations of *Brucella*-like lesions on Victoria Island, Canada (Tomaselli et al. 2016). We are just starting to recognize the extent and importance of disease in muskox population dynamics. To provide information on the prevalence, significance, and role disease plays in muskox population dynamics, we acknowledge the need to adopt standardized health assessment protocols, systematically document local knowledge on muskox health, and the use of more advanced modeling methodologies. Subsequent development of assessments for general population health would complement surveys for abundance. The Electronic Supplementary Materials (Tables S1, S2) provide an up-to-date overview of pathogens and diseases described in muskoxen.

## Drivers and knowledge gaps

The vulnerability and resilience of muskoxen and associated knowledge gaps were discussed extensively at the 2016 muskox health ecology symposium (Kutz et al. 2017). Here, we define a driver as a major change that generates stressors. We regard stressors as typically regional events or conditions that create impacts locally for specific populations. These impacts bring about changes in populations, including demographics, movement and dispersal patterns, health. The CBMP Freshwater group identified climate and human activity as the most influential factors changing the hydrology, pollutions, and biochemistry of regions (Lento et al. 2018), all of which will affect herbivores, including muskoxen.

## Climate change

The consequences of climate change on life in the Arctic are diverse, multifaceted, and largely unknown. We summarize here stressors and effects with the greatest potential to alter muskox population dynamics.

### Stressors: Stochastic events and weather extremes

For over half a century, changes in calf productivity and survival have been linked to annual variability in regional weather patterns (Tener 1965; Miller and Russell 1975). Increasing temperatures, especially in fall and winter, increase the likelihood of extreme weather events including deeper than average snow depths (Gunn et al. 1989; Reynolds 1998), ice-crust formation (Forchhammer and Boertmann 1993), and rain-on-snow events (Gunn et al. 1989; Putkonen et al. 2009). All can reduce feed availability and increase the energetic cost of foraging, which may lead to increased mortality and decreased calf recruitment (Parker et al. 1975; Gunn and Adamczewski 2003; Miller and Barry 2009). Analyses of long-term datasets reveal a more complex and less predictable association between winter precipitation, ice-crust formation, and muskox population dynamics (Forchhammer and Boertmann 1993; Schmidt et al. 2015). This reinforces the importance of considering the impact of both temporal and spatial scale on interpretations of individual studies and datasets (Post et al. 2009; Bölter and Müller 2016). Examples include the regional-scale decline in muskox abundance, of more than 90%, after three consecutive winters of record snowfall in the Bathurst Island Complex (Miller 1998), and on a smaller spatial and temporal scale, the Alaskan tidal surge which entombed 55 muskoxen in ice (Adams in Kutz et al. 2017; Berger et al. 2018). The impact of increasing frequency, distribution, severity, and extent of stochastic events on population dynamics remains a serious knowledge gap for this species.

Muskoxen are well adapted to life in cold, dry habitats and there is a tendency to think of cold environments as essential to their survival. However, there is wide thermal variability within their endemic habitat (mean summer maximums of 21°–27 °C to mean winter minimums of − 34 °C: Tener 1965). On the Canadian Arctic mainland, muskoxen are currently extending their range southward (Adamczewski in Kutz et al. 2017), and translocated animals (both captive and wild) have survived in a variety of habitats both warmer and wetter than their traditional range (Lent 1999). There are currently seven muskox populations living in CAFF’s designated Sub Arctic Zone, and a further two that live in non-arctic zones (Fig. 1, Table 1). Local conditions, like availability of shade, shallow water for wading, and snow patches, may mitigate the effects of warm ambient temperatures (Cuyler pers. comm.). Regardless, increases in heat and humidity can precipitate serious adverse effects, especially when these co-occur with other stressors (e.g., pathogens, nutrient deficiencies, disturbance, and predation) or during sensitive periods (i.e., calving, rut) (Ytrehus et al. 2008, 2015). Shifts in temperature and precipitation regimes are predicted for the Arctic, and carry the possibility of influencing muskox reproduction and survival.

### Impacts: Changing vegetation, species associations, and disease

Changing vegetation diversity, abundance, composition, and phenology in the Arctic are all well documented (Sturm et al. 2001; Walker et al. 2006; Bjørkman et al. 2020). Landscape-scale changes in vegetation (e.g., shrubification), affect ecosystems at multiple trophic levels (Myers-Smith et al. 2011; Mod and Luoto 2016) and have generated concerns about trophic mismatch (Kirby and Post 2013). Before we can address the effects of climate change on forage quantity and quality, we need to understand the impact of normal grazing on these matrices under differing animal densities and at multiple scales. Muskox grazing can alter carbon dioxide and methane fluxes (Falk et al. 2015), redistribute nutrients (Murray 1991; Mosbacher et al. 2016), alter plant community composition (Mosbacher et al. 2018), sometimes mitigate shrubification (Post and Pedersen 2008), and enhance graminoid production (Mosbech et al. 2018). In addition to vegetation biomass, an understanding of the complete nutrient value of the vegetation and its correlation with population health is currently lacking. Trace mineral deficiencies in wild ruminants predispose them to a range of subclinical ailments including poor reproductive performance, immunosuppression, and anemia (Blake et al. 1991; Afema et al. 2017), all of which makes them more vulnerable to pathogens, predation, and weather. Monitoring programs need to incorporate a clear, unified criterion for defining and evaluating grazing disturbances on vegetation at multiple temporal and spatial scales. Establishing baseline reference ranges for the complete nutrient value (including an approximate range of possible year-to-year variations) of muskox forages throughout the north is an essential compliment to these data.

Changes in temperature and precipitation are likely to influence the trophic context faced by muskoxen, not just from changes in vegetation, but potentially from mosquitoes and other biting insects. Although the role of insect harassment on caribou ecology is relatively well documented (Raponi et al. 2018), their role in muskox ecology is not. Simultaneously, the northward expansion or changing densities of species, ranging from potential predators to herbivore competitors or species capable of altering ecosystems (e.g., beaver *Castor canadensis*: Tape et al. 2018) is unprecedented in our time and presents unknown, unevaluated risks and/or benefits. Historically, wolves (*Canis lupus*) were considered the main predator in muskox ecosystems (Marquard-Petersen 1998; Gunn and Adamczewski 2003; Mech 2011). Now, documentation of grizzly bear (*Ursus arctos*) predation, originally considered a sporadic occurrence, is increasing in some regions (Gunn and Adamczewski 2003; Arthur and Del Vecchio 2017). Grizzly bears are a more important predator than wolves in northeastern Alaska (Reynolds et al. 2002). Information on muskox predator–prey relationships, especially in multi-prey situations, is necessary to understand and predict population trends.

Changing patterns of infectious and non-infectious disease have been documented across several muskox populations in the last decade. Climate warming is behind some changes, while causes in other instances are less well understood. Through morbidity, reproductive failure, and mortality, pathogens, whether introduced or endemic, are likely to play a role in changing the distribution and dynamics of muskox populations. Furthermore, none of the specified stressors is acting in isolation. Ultimately, environmental and nutritional factors may be enabling infectious agents to cause overt disease, or alternatively subclinical disease, which may predispose individuals to a host of stressors, and through complex interactions determine the cumulative impact on muskox population dynamics.

## Anthropogenic change

A consequence of warming temperatures in the Arctic is the overall increase in human activity, especially in previously inaccessible habitats. Predicting how muskoxen will respond to the greater human presence is difficult.

The impact of increasing industrial pursuits (oil and gas, open pit mines), as well as their associated pollutants (Gamberg and Scheuhammer 1994) or pollutants accumulating from more southern locations (Salisbury et al. 1992), need to be documented and monitored, especially considering the role of muskox in subsistence food economies.

Today’s greater access to a previously remote Arctic has also contributed to the increasing appeal of the Arctic as a tourist destination. While expanding tourism provides new economic opportunities to northern residents, it is also associated with serious challenges, including but not limited to, environmental degradation and increasing problems with waste disposal and pollution from greater ship and air traffic (CAFF 2013).

At the local community level, climate change in the Arctic has sometimes made areas less predictably accessible depending on the season (Kutz pers. comm.), while elsewhere opportunities to access remote terrain have expanded with modern modes of transportation, and contributed to a proliferation of summerhouses and year-round use (Cuyler pers. comm.).

Food insecurity in northern communities is a growing concern and a significant public health problem (Ruscio et al. 2015). With decreasing access to subsistence and traditional foods, northern communities are seeking sustainable alternatives. Some are considering or have begun implementing agricultural practices, including livestock production (Caviezel et al. 2017). Livestock creates a new source of competition for muskox food resources, and avenues for the introduction of novel pathogens.

Hunting contributed to the muskox decline of the early 1900s (Lent 1999). Muskox harvesting, whether strictly for subsistence or for broader commercial enterprises, must be carefully monitored and sustainable yields enforced. Today, most, but not all, muskox harvests are regulated. Enforcement, however, is often a difficult task, owing to large uninhabited areas, insufficient resources and people (e.g., six hunting officers for all of Greenland; Cuyler pers. comm.). Levels of hunter compliance are not well known. Recently, global markets for muskox qiviut wool, also known as ‘Arctic Gold,’ have grown rapidly (Jørgensen 2019). The low availability of qiviut relative to current demand has driven prices up sharply for raw winter skins and ultimately qiviut wool (Jørgensen 2019). For hunters, this has created opportunities for large instant profits. Although illegal in Greenland, killing muskoxen for just their winter skins, and out-of-season harvesting using prohibited methods occurs (Cuyler pers. comm.) Assuming global demand for qiviut wool will continue rising, even vigilant monitoring and enforcement may not be enough to ensure continued sustainable use of present muskoxen populations. The new market situation may require regulation of the trade in muskox skins. Simultaneously, reliable harvest data are scarce, making it difficult to document the numbers of muskoxen taken or the economic contribution to northern communities. Further, depending on the type of harvest, it may affect muskox group composition and ultimately population dynamics (Rockwood 2015), yet an assessment of effects on muskox abundance and demographics is difficult without reliable harvest data. We also generally lack effective user-friendly models to determine sustainable harvesting levels and thresholds (Cuyler pers. comm.). The concept of adaptive management (Madsen et al. 2017) might be a suitable platform to help ensure appropriate regulations development, while taking into account all stakeholders. A market economy can drive population changes, either by exerting a negative downward pressure (Berger et al. 2013), or by encouraging northern communities to consider the economic potential, and thus bolster conservation efforts. Developing strategies to facilitate cooperative management between agencies and local communities will foster the latter outcome, e.g., the PISUNA (2014) initiative as implemented in Greenland.

## Key findings and next steps

This is the first summary containing current information for all muskoxen populations. Recognizing the limitations inherent in these data, we estimate global abundance of muskoxen at ca 170 000. Climate, diseases, and anthropogenic changes, singly or any interaction thereof, constitute the major foreseeable challenges for muskoxen. Which elements become critical for a specific population will vary and depend on a host of local interacting variables, which may be difficult to predict or mitigate, e.g., stochastic weather events.

There is an acute need to increase the frequency of surveys and standardize the variety of existing monitoring protocols, including consistent definitions and methodology for how survey areas and range limits are determined, especially how populations are defined. We need more data and standardized protocols on demographics and harvest specific to each population. Wherever possible, new monitoring initiatives must include health assessment metrics, local weather events, and increased traditional knowledge contributions.

The most effective path forward is to leverage existing resources. Multidisciplinary approaches will enable the most rapid gains in the shortest period. Using MOXNET membership, collaborative initiatives can be developed regionally and internationally to address the next steps.

Establishing standardized protocols can begin by building on recognized practices such as those developed by the CARMA network for caribou (CircumArctic *Rangifer* Monitoring & Assessment) (Gunn and Russell 2008; Gunn and Nixon 2008; Kutz et al. 2013a). Further development would incorporate new, innovative approaches for monitoring health and disease, include integration of traditional ecological knowledge and community-based monitoring, and expand scope and range with emerging technologies (Kutz et al. 2017). To be effective these protocols must incorporate from inception to implementation, local input through strategies such as co-management programs, hunter participation, and local knowledge (Tomaselli et al. 2018a, b).

While MOXNET is an organization with a primary focus on muskoxen, multidisciplinary input is necessary to incorporate an ecosystem approach, e.g., abiotic monitoring, specifically the intensity and extent of adverse weather events; monitoring changes in vegetation and the impact of grazing at multiple temporal and spatial scales; monitoring the impact of changing species’ boundaries on predator/prey relationships. Only through an interdisciplinary lens can we identify and exploit existing opportunities. For example, the low genetic diversity and widespread translocations/re-introductions of muskoxen around the Arctic create the opportunity of almost unprecedented investigations into the plasticity of muskox traits (morphological, phenological, behavioral, etc.) relative to a variety of environmental conditions, all while holding evolutionary history as a constant.

Finally, we need to facilitate data sharing with a collaborative focus on the establishment of a circumpolar database, its infrastructure, and management. This will enable the harmonization of existing data sources, feed into the creation of predictive models, and prioritize future research directions.

## Electronic supplementary material

Below is the link to the electronic supplementary material.

Supplementary material 1 (PDF 1260 kb)

Supplementary material 2 (XLSX 88 kb)
