# Supplementary material for: Muskox status, recent variation, and uncertain future
Source: Ambio. 2019 Jun 11;49(3):805–19. doi: 10.1007/s13280-019-01205-x (PMC6989413; doi:10.1007/s13280-019-01205-x)
Supplement: Supplementary file 1 — Supplementary material 1 (PDF 1260 kb) [file 13280_2019_1205_MOESM1_ESM.pdf]

Ambio
Electronic Supplementary Material
This supplementary material has not been peer reviewed

Title: **Muskox status, recent variation, and uncertain future**

Christine Cuyler, Janice Rowell, Jan Adamczewski, Morgan Anderson, John Blake, Tord Bretten, Vincent Brodeur, Mitch Campbell, Sylvia L. Checkley, H. Dean Cluff, Steeve D. Côté, Tracy Davison, Mathieu Dumond, Barrie Ford, Alexander Gruzdev, Anne Gunn, Patrick Jones, Susan Kutz, Lisa-Marie Leclerc, Conor Mallory, Fabien Mavrot, Jesper Bruun Mosbacher, Innokentiy Mikhailovich Okhlopkov, Patricia Reynolds, Niels Martin Schmidt, Taras Sipko, Mike Suitor, Matilde Tomaselli, Bjørnar Ytrehus

Muskoxen: Past and present

We begin with brief backgrounds on muskox evolution and early history, ecosystem services, general biology and known diseases and parasites. We finish with a circumpolar summary of all wild muskox populations by country and region, as well as extirpations. A history of surveys for monitoring muskoxen population size are in online supplementary materials excel Table S3.

Muskox Evolution and Early History

From their Asian origin, muskoxen arrived in Europe nearly 1 million years ago, moving into North America ca 250,000-150,000 years ago. Although widespread across Arctic Siberia and North America, muskox numbers were low compared to Pleistocene herbivores that shared their environment e.g. bison (*Bison spp*.), horses (*Equus spp.*), mammoth (*Mammuthus spp.*) and reindeer (*Rangifer tarandus spp.*) (Campos et al. 2010). The previously rich diversity of fauna declined through the Last Glacial Maximum and into the Holocene, and by the late Pleistocene over 35 genera had gone extinct (Webb and Barnosky 1989; Grayson and Meltzer 2002), including several grazing herbivores (Guthrie 2001). This period of extinction (13,000-11,000 years BP) coincided with a warming climate and expanding refugia (Pielou 1991; Shapiro et al. 2004; Guthrie 2006; Campos et al. 2010). With little evidence to support human harvest as a primary cause of faunal extinctions (Grayson and Meltzer 2002), attention focused on the major shift in climate patterns. Changing climate altered the vegetation from a steppe-like graminoid tundra to a wetter regime supporting woody shrubs and mosses (Guthrie 2001, 2006). Muskoxen were among a handful of species that survived. Today, muskoxen are the only living member of the genus *Ovibos* (Tribe Ovibovini, Subfamily Caprinae; Simpson 1945), a classification that acknowledges their close physiological and anatomical affinity with goats. The gorals (*Naemorhedus spp.*) of Central and East Asia are their nearest living relatives (Groves, 1997).

Muskoxen have been through several near extinctions and extirpation events. They disappeared from Europe 10,000 years ago (Campos et al. 2010), vanished from Russia 2000-1000 years ago, and by the late 1800’s, were gone from Alaska and northwestern Canada (Lent 1999). Extant populations existed only in northern Canada and Greenland (Tener 1965; Lent 1999). Populations continued to decline throughout Canada during the late 1800’s into the first decades of the 20th century, concurrent with increasing commercial harvests for muskox hides (Barr 1991). In east Greenland, they were exploited to feed whalers, sealers, trappers, explorers and farmed foxes, and to obtain young animals for zoos and translocations (Jennov 1941; Lent 1999); yet they survived, although not without a cost. Current genetic data supports historical and archeological evidence (MacPhee et al. 2005) suggesting muskoxen have gone through multiple ‘bottlenecks’ during their evolution, resulting in a species that is challenged by low genetic variability (Gordeeva et al. 2009; Cooley et al. 2011; Thulin et al. 2011; Hansen et al. 2018). In the past, a few surviving local pockets may have been responsible for this species continued existence.

As concerns grew about declining numbers of muskoxen in the first decades of the 20th century, conservation efforts arose. In Canada, restrictions on trading muskox hides passed in 1917, and was followed in 1927 by the creation of the Thelon Game Sanctuary, intended to protect remnant populations of muskoxen from hunting (Tener 1965). Conservation efforts came more slowly to Greenland, being complicated by issues of sovereignty. Simultaneously, the idea of introducing muskoxen to different circumpolar regions gained popularity. For some this was a means of conserving the species, while for others the prospect of domesticating muskoxen was the motivating force. Throughout the 1920’s and 30’s East Greenland muskoxen were translocated to Svalbard, Dovre Mountains and other parts of Norway and to Alaska. The thirty-one Greenland muskoxen taken to Nunivak Island in Alaska ultimately became the founding stock for seven muskox populations today, six

in Alaska and one in the Yukon. The history of early conservation and translocation efforts is summarized in Lent (1999).

Ecosystem services

Muskoxen are emblematic of the Arctic, a symbol of resilience and survival against the odds. As non- hibernating/migrating large herbivores, they influence all trophic levels within the Arctic ecosystem by their year round presence. Grazing enhances plant productivity and plant species composition (Murray 1991; Raillard 1992; Post et al. 2009; Mosbacher et al. 2016) ultimately enriching underlying soils and affecting freshwater community diversity. Winter grazing exposes patches of vegetation facilitating access for other species, (Gray 1987). As herbivores, they are prey for large carnivores including humans, while their carcasses provide food and shelter for scavengers (Schmidt et al. 2008).

General Biology

Their northern name, umingmak / ᐅᒥᖕᒪᒃ (Inuinnaqtun/Inuktitut, Canada), umimmak (Greenland) translates roughly to the “bearded one”, a reference to their distinctive long hair. In contrast, the common name muskox was based on the assumption that they were a type of ‘oxen’ and their characteristic odor came from musk glands, neither of which is true.

Muskoxen have short stocky bodies. Long, coarse outer guard hair hangs almost to the ground. This protection from the elements also traps the heat within the thick, dense layer of fine under wool (qiviut), which completely covers the body from ears to tail. Muskoxen consume nutrient rich forages, graminoids and willows, where available (Tener 1965; Obrien 1988; Klein and Bay 1990; Raillard 1992; Kristensen et al. 2011), building up fat reserves during the short summer season. In winter, their food intake decreases and they maintain body mass on reduced amounts of lower quality forage (Adamczewski 1994). This, coupled with decreased movements and activity, further conserves energy (Reynolds 1998; Schaefer and Messier 1995a, b, 1996). Muskoxen preferentially graze in areas of shallow snow where they can paw through the snow crust and gain access to sedges and dried forbs (Wilson 1992; Schaefer and Messier 1995b). In years of deep snow, they move to wind-blown ridges where willow tends to dominate the diet (Klein and Bay 1990, 1991; Rozenfeld et al. 2012; Mosbacher et al. 2016a, b).

Group size and composition varies seasonally. In winter, females, sub-adults and some males aggregate into large mixed groups, while many males are in small bull groups. During late summer into the rut, dominant males maintain small harems, and non-breeding males are often solitary. Groups typically change in size and membership as they merge and split apart (Reynolds 1993).

Muskoxen are seasonal breeders, breeding in August-September throughout most of their range (Tener 1965; Gray 1987). Females are polyestrous when in season with an average estrous cycle length of 20 days (range: 19-21 days, Rowell and Flood 1988). Gestation length is approximately 8 months (236-250 days: Alendal 1971; Rowell et al. 1997, 2007) and culminates with the birth of a single calf. Twinning is rare although it has been documented (Alendal 1971; Wilkinson 1971). Reports of dystocia (calving difficulties, Wilkinson 1971) and freemartins (masculinization of a female fetus co-twin with a male) associated with twinning among captive muskoxen reinforces the conclusion that twinning is not a strategy for population growth in this species. Similar to other ruminants, onset of puberty depends on body weight and therefore varies across their range. The probability of successful breeding increases with female body mass (Rowell et al. 1997; White et al. 1997). Captive cows can produce calves at age 2-years (Rowell 1991) and, under favourable conditions, may result in rapidly expanding wild populations (Alendal 1971; Jingfors and Klein 1982; Tessaro et al. 1984; Olesen 1993; Reynolds 1998). However, first calf production at age 3-years is more common in most habitats (Schmidt et al. 2015; Tessaro et al.

1984; Latour 1987; Reynolds 2001). The duration of lactation is variable and can extend into mid-winter or later (Rowell et al. 1997; White et al. 1997). Lactation does not necessarily interfere with successful breeding and females in good body condition can calve annually (White et al. 1997), a phenomenon again associated with expanding populations (Jingfors and Klein 1982). Poor female body condition can result in non-annual breeding (White et al. 1997). The amount of body fat associated with a 50% probability of pregnancy in muskox cows (age ≥ 3-years) is 22%, which is much higher than for caribou (Adamczewski et al. 1998). In wild populations, females calve at intervals of 2-3 years, presumably because they failed to regain body reserves during a single summer season after pregnancy and lactation (Reynolds 2001). Other cause(s) of low calf:cow ratios are not always evident. Nonetheless, the ratio of calves per 100 cows (age ≥ 3-years) in summer (annual productivity) or in late winter (calf recruitment) are frequently the most useful indicators of how a population is trending.

Proximate causes of mortality among free-ranging muskoxen include starvation in years of deep snow or icing (rain-on-snow) events, predation by wolves (*Canis lupus)* and grizzly bears (*Ursus arctos*), disease and hunter harvest (Gray 1987; Blake et al. 1991; McLean et al. 1993; Reynolds et al. 2002; Kutz et al. 2004, 2015; Afema 2008; Grenfell and Putkonen 2008; Vikøren et al. 2008; Ytrehus et al. 2008, 2015; Schmidt et al. 2008; Rennert et al. 2009; Forde et al. 2016).

Disease & Parasites

Disease influences wildlife populations (Berger et al. 1998; Blehert et al. 2009; Kock et al. 2018) but alone is unlikely to lead to species extinctions or even declines (Smith et al. 2006; Pedersen et al. 2007). The large-scale outbreak of *Y. pseudotuberculosis* on Banks Island was not associated with a declining population but rather a continued rise in muskox numbers (Blake et al. 1991). In contrast, the bacterium *Erysipelothrix rhusiopathiae* might have been a contributing factor to the population decline in Banks Island muskoxen (Kutz et al. 2015). The multifaceted impact of disease on host populations (e.g. direct mortality, reduced fitness and reproduction) makes it extremely difficult to evaluate (Preece et al. 2017; Kock et al. 2018). It is common to focus on specific infectious agents as a cause of disease. However, nutritional status and environmental factors may be key drivers enabling infectious agents to cause overt disease, as well as subclinical disease, which plays a more insidious role, predisposing individuals to a host of stressors. Not all diseases come from new host-pathogen interactions (Kutz et al. 2004). The parasite *U. pallikuukensis* and gamma herpes virus (Type 1 Ruminant Rhadinovirus) are specific to muskoxen (Kutz 1999; Kutz et al. 1999a,b; Handeland et al. 2018) suggesting a degree of coevolution and a potential role for disease in past muskox declines. Immune function is an animal's first and primary defense against disease and immune suppression contributes to the severity and spread of disease. A study in captive muskoxen demonstrated passive transfer of immunity was effective and equivalent to that of domestic livestock (Rosa et al. 2007). Additionally, a recent investigation of the function of large granular lymphocytes provides an initial step in characterizing immune function as well as an additional tool for future studies on muskox population health (Desforges et al. 2018).

We tabulate (TS1, TS2) the growing list of pathogens, parasites and disease conditions identified in muskoxen with the associated references. Different diagnostic tools enable researchers to map pathogen occurrence on both a temporal and spatial scale: serology (detection of antibodies in the serum) informs on past exposure whereas direct detection technics (culture, Polymerase Chain Reaction [PCR]) provide insight on current infection status. Full necropsies of both sick and apparently healthy animals enable the identification of health issues and help detect other co-morbidity factors. Furthermore, bioinformatics (strain analysis, whole genome sequencing) provide additional information on the epidemiology and pathogenicity of infectious agents. A circumpolar systematic and holistic approach to health assessment and disease investigation in muskoxen is necessary if we hope to understand the predisposing conditions that precipitate clinical disease and the possible role of disease in muskox population fluctuations.

Table S1 Infectious and non-infectious diseases reported in muskoxen.

Disease Category	Etiology	Pathology reported in muskoxen	Reference
**Infectious**			
**bacterial**	*Yersinia pseudotuberculosis*	Ulcerative enteritis-typhlitis and sepsis	Blake & McLean 1988; Blake et al. 1991; McLean et al. 1993; McLean 1996
	Brucella suis biovar 4	Joint disease, bursitis, abscesses	Gates et al. 1984; Forbes 1991; Tomaselli et al. 2016; Tomaselli 2018.
	Erysipelothrix rhusiopathiae	Sepsis	Kutz et al. 2015
	Pasteurellaceae spp.	Bronchopneumonia	Ytrehus et al. 2008
	Mycoplasma ovipneumoniae	Bronchopneumonia	Handeland et al. 2014; Afema et al. 2017
	Chlamydophila spp	Joint disease	Afema et al. 2017
	Bibersteinia trehalosi	Bronchopneumonia	Afema et al. 2017
	Trueperella (Corynebacterium) pyogenes	Bronchopneumonia, Abscesses	Beckley & Dieterich 1970; Afema et al. 2017
**viral**	*Parapoxvirus* (orf virus)	Proliferative and pustular dermatitis	Falk 1978; Kummeneje & Krogsrud 1978; Dieterich et al. 1981; Zarnke et al. 1983; Jørgensen et al. 1984; Mathiesen et al. 1985; Tomaselli et al. 2016; Afema et al. 2017; Tryland et al. 2018
	Gamma herpes virus	None reported	Zarnke et al. 2002; Li et al. 2003; Vikøren et al. 2013; Handeland et al. 2018
	Coronavirus	None reported	Das Neves et al. 2016
	Respiratory syncytial virus	None reported	Das Neves et al. 2016
	Ruminant pestivirus	None reported	Das Neves et al. 2016
	Parainfluenza virus type 3	None reported	Das Neves et al. 2016
**Non-infectious diseases**			
	Copper deficiency		Blakley et al. 2000; Barboza & Blake 2001; Rombach 2001; Swor 2002; Barboza et al. 2003; Rombach et al. 2003
	Malnutrition/Emaciation		Ott 1998; Afema et al. 2017
	Incisor Breakage		Kutz et al. 2017; Mavrot et al. 2017

Table S2 Summary of known parasites in muskoxen populations.

Family	Species name provided	Reference
**Cestodes**	Cysticercus (bladder phase) of tapeworm	Jensen 1904
	Cysticercus tenuicollis	Dikmans 1939; Gibbs & Tener 1958
	Echinococcus granulosus (Hydatid disease)	Gibbs & Tener 1958; Seidel & Rowell 1996
	Moniezia sp.	Tener 1954; Spencer & Lensink 1970; Samuel & Gray 1974; Alendal & Helle 1983; Seidel & Rowell 1996; Ott 1998; Davidson et al. 2014
	Moniezia expansa	Tener 1954; Durrell & Bolton 1957; Gibbs & Tener 1958; Tener 1965; Webster & Rowell 1980; Korsholm & Olesen 1993; Ott 1998
	Taenia sp.	Feilden 1877; Lønø 1960
	Taenia hydatigena cysticercus	Jensen 1904; Dikmans 1939; Gibbs & Tener 1958; Webster & Rowell 1980; Alendal & Helle 1983; Ott 1998
**Nematodes**	*Capillaria* sp.	Samuel & Gray 1974
	Chabertia ovina	Alendal & Helle 1983
	Cooperia oncophora	Alendal & Helle 1983
	Dictyocaulus sp.	Beckley & Dieterich 1970; Alendal & Helle 1983; Davidson et al. 2014
	Dictyocaulus eckerti	Kutz et al. 2012
	Dictyocaulus viviparus	Durrell & Bolton 1957; Gibbs & Tener 1958; Samuel & Gray 1974; Alendal & Helle 1983; Seidel & Rowell 1996; Ott 1998
	Filaria sp.	Feilden 1877
	Grosspiculagia occidentalis	Bye et al. 1987
	Haemonchus contortus	Canavan 1929; Durrell & Bolton 1957
	Marshallagia sp.	Samuel & Gray 1974; Alendal & Helle 1983
	Marshallagia marshalli	Dikmans 1939; Webster & Rowell 1980; Alendal & Helle 1983; Bye et al. 1987; Korsholm & Olesen 1993; Ott 1998
	Muellerius capillaris	Davidson et al. 2014
	Nematodirella sp.	Samuel & Gray 1974
	Nematodirella alcidis	Kutz et al. 2012
	Nematodirella gazelli	Kutz et al. 2012
	Nematodirella longispiculata	Gibbs & Tener 1958; Samuel & Gray 1974
	Nematodirella longissimespiculata	Alendal & Helle 1983; Korsholm & Olesen 1993
	Nematodirus sp.	Samuel & Gray 1974; Korsholm & Olesen 1993; Davidson et al. 2014
	Nematodirus filicollis	Alendal & Helle 1983
	Nematodirus helvetianus	Samuel & Gray 1974; Webster & Rowell 1980; Alendal & Helle 1983; Korsholm & Olesen 1993; Ott 1998
	Nematodirus skrjabini	Alendal & Helle 1983
	Nematodirus spathiger	Alendal & Helle 1983

	Nematodirus tarandi	Kutz et al. 2012
	Ostertagia sp.	Samuel & Gray 1974; Alendal & Helle 1983; Seidel & Rowell 1996
	Ostertagia arctica	Alendal & Helle 1983
	Ostertagia circumcincta	Dikmans 1939; Gibbs & Tener 1958; Alendal & Helle 1983; Korsholm & Olesen 1993
	Ostertagia gruehneri	Alendal & Helle 1983; Bye et al. 1987
	Ostertagia marshalli	Dikmans 1939
	Ostertagia occidentalis	Dikmans 1939; Alendal & Helle 1983
	Ostertagia trifurcata	Webster & Rowell 1980; Alendal & Helle 1983; Korsholm & Olesen 1993
	Protostrongylidae (not identified)	Hoberg et al. 2002; Davidson et al. 2014
	Protostrongylus stilesi	Kutz et al. 2012
	Skrjabinagia arctica	Bye et al. 1987
	Teladorsagia boreoarcticus	Hoberg et al. 1999
	Teladorsagia circumcincta	Bye et al. 1987; Ott 1998
	Teladorsagia davtiani	Alendal & Helle 1983; Korsholm & Olesen 1993; Ott 1998
	Teladorsagia trifurcata	Bye et al. 1987; Ott 1998
	Trichostrongylus axei	Alendal & Helle 1983
	Trichostrongylus capricola	Alendal & Helle 1983
	Trichostrongylus columbriformis	Alendal & Helle 1983
	Trichostrongylus vitrinus	Alendal & Helle 1983
	Trichuris sp.	Samuel & Gray 1974; Alendal & Helle 1983
	Trichuris ovis	Canavan 1929; Seidel & Rowell 1996
	Umingmakstrongylus pallikuukensis	Hoberg et al. 1995; Kutz et al. 2013; Tomaselli et al. 2016
	Varestrongylus eleguneniensis	Kutz et al. 2013; Verocai et al. 2014; Kafle et al. 2017
**Trematodes**	*Fascioloides magna*	Lair et al. 2016
**Protozoan**	*Besnoitia spp.*	Leighton & Gajadhar 2001
	Cryptosporidium spp	Davidson et al. 2014
	Eimeria spp.	Duszynski et al. 1977; Seidel & Rowell 1996; Davidson et al. 2014
	Giardia duodenalis	Davidson et al. 2014
	Sarcosystis spp.	Samuel et al. 1974
	Toxoplasma gondii	Kutz et al. 2000

Muskox status & trends around the Arctic

The recent historic (1800s) distribution of endemic muskoxen was limited to North America and Greenland. They occurred from Point Barrow Alaska (USA) across Canada's northern territories even penetrating into the northeastern portion of Manitoba province, finally reaching across the north of Greenland and down its northeast coast. However, this distribution shrank by the turn of the 20th century, when several populations disappeared or declined in North America (Lønø 1960, Grubb 2005). Today endemic muskox populations remain in Canada and Greenland (IUCN 2018). In Canada, muskoxen inhabit most of the large Arctic islands (except Baffin) and are widespread over the mainland tundra of the Northwest Territories and Nunavut. Endemic distribution in north and northeast Greenland is essentially unchanged. Present day distribution has grown through the expansion of endemic populations and numerous translocations to regions of former range and previously unoccupied regions. Although translocations were sometimes unsuccessful, some resulted in rapid population growth (e.g., Alaska, Reynolds 1998; West Greenland, Olesen 1993) and recolonization of historic muskox ranges. Muskox density varies widely around the Arctic (Online supplementary materials, Excel Table S3). Better habitat quality should permit greater density, but multiple factors are likely involved, including harvest, predation, disease and local weather conditions.

Muskox status & trends by country

Alaska

Where not specifically cited, the information below is from personal communication with Patrick Jones, Elizabeth Lennart and Patricia Reynolds.

Endemic muskoxen disappeared from Alaska by the 1890's. Re-introduction began when 31 muskoxen (*O.m. wardi)* captured in 1930 on the East coast of Greenland were shipped to the Alaska Agricultural College near Fairbanks and released 5 years later on Nunivak Island on the western Alaska coast (Lent 1999). By the 1960's and 1970's, the population on Nunivak was large enough to permit the translocation of muskoxen to four other locations: Nelson Island, near the Yukon Kuskokwim delta where animals could move to and from the mainland, Seward Peninsula in western Alaska, Cape Thompson in northwestern Alaska, and near the Arctic National Wildlife Refuge in northeastern Alaska. Initially the re-introduced populations grew in abundance and all exhibited range expansion, except Nunivak Island from which animals could not easily disperse. Animals from Nelson Island moved onto the mainland creating a population on the Yukon Kuskokwim Delta. Muskoxen in the Arctic National Wildlife Refuge (original North East population) expanded their range both west and east eventually vacating areas first occupied and splitting the population into two segments in north-central Alaska (North East population) and northwestern Yukon, Canada (Yukon North Slope population) (Reynolds et al. 2011).

Annual surveys for North East muskoxen took place from 1982 through 2018 in the Arctic National Wildlife Refuge and adjacent areas to the west (Reynolds 2006; Reynolds 2011; Lenart 2015). Annual monitoring also occurred on Nelson and Nunivak Islands where subsequent harvest adjustments kept muskox abundance on these islands stable. Population distribution is based on the presence of mixed groups of muskoxen (females, sub-adults and often one or more adult males). Solitary males and small groups of males are often seen long distances from mixed groups. Seward Peninsula surveys commonly used distance sampling (Schmidt et al. 2010). Numbers in the North East population initially increased rapidly, and then declined by about 50% from 1995-2005, stabilizing around 200 muskoxen in 2006-2011. Today this population appears to be increasing, as the 2018 pre-calving survey counted 285 muskoxen. These numbers are below the historic highs in Reynold (2011). Previously, illegal harvest

limited growth of the Yukon Kuskokwim Delta muskoxen. By 2018, however, this population appears to be growing. The current conservative estimate for muskoxen in Alaska is ca 4,300, which is 2.6% of the global total.

Canada

Canada is the only country to have endemic populations of both *O.m. wardi* and *O.m. moschatus*. Excepting the Yukon, the former are generally found on the Arctic Archipelago, and the latter on the mainland. The Canadian Arctic islands fall under the jurisdictions of either the Northwest Territories (NT) or Nunavut (NU). Details for island areas and groupings are in the Management Plan for High Arctic Muskox in the Qikiqtaaluk Region 2013-2018, and in Jenkins et al. (2011). The NT contains five administrative regions. Furthest north is Inuvik, which includes both Arctic islands and mainland. The Sahtu, North Great Slave, South Great Slave and Dehcho comprise mainland NT. The NU contains three administrative regions, Kitikmeot, Kivalliq and Qikiqtaaluk. Current estimate for total number of muskoxen in Canada is ca 109,000, which is 64.6% of the global total.

Yukon Territory (Suitor pers. comm.)

The historical coastal range of the Yukon North Slope was re-colonized by natural expansion of *O.m. wardi* muskoxen from Alaska's North East population, when the latter still occupied Alaska's Arctic National Wildlife Refuge, which is adjacent to the Yukon. Immigration is known to have occurred ca 1985-1986 and again in 1999-2001. Range expansion has since been eastward along the Yukon coast and southward. In recent years, large mixed groups have established themselves inland in the British, Barn and Richardson mountains. There are also groups west of the Mackenzie Delta in the Northwest Territories (NT). There are frequent sightings of single bulls or small bull groups south of the Arctic Circle, even within the vicinity of Dawson City (64° N). Since the early 2000's the population has likely increased by ca 50%. Monitoring of the Yukon North Slope population in the 2013-2018 period, observed high calf productivity (i.e., midsummer ratios of ≥ 40 calves/100 cows). In 2018, the Yukon and adjacent area west of the Mackenzie Delta in NT had a growing population of ca 344 muskoxen above the tree line.

Northwest Territories mainland

Despite near extirpation in the early 1900s. Muskoxen on the NT mainland have recolonized their historic range and are now south of the tree line. Although no muskoxen are known in the Dehcho, the Sahtu, North Great Slave, South Great Slave regions have experienced an expansion of their endemic *O.m. moschatus* muskoxen, which suggests population growth. In contrast, the mainland Inuvik muskoxen appear to be stable. Anecdotal observations are frequent and increasing in the South Great Slave region (no. 11 Fig 2, Fig S1), but surveys have yet to provide an estimate of abundance. Muskoxen have been sighted below 60˚ N. Lat. in northern Alberta and Saskatchewan; however, their extent is unknown. Greater detail regarding muskox distribution within the current NT administrative units will be available in Gunn et al. (in prep). Declines may have followed some expansions. Abundance, distribution and population trend for the NT are obscure because some areas have never been surveyed, and others infrequently. For example, the 1997 muskox survey (Sahtú region, Veitch 1998) covered a large area north of the tree line; since then muskoxen have expanded south into forested areas, but no further regional muskox surveys have occurred. The recent 2018 survey of the North Great Slave region, meanwhile, estimated ca 8,100 muskoxen (Cluff pers. comm.). Today, muskoxen on the NT mainland may total ca 12,574.

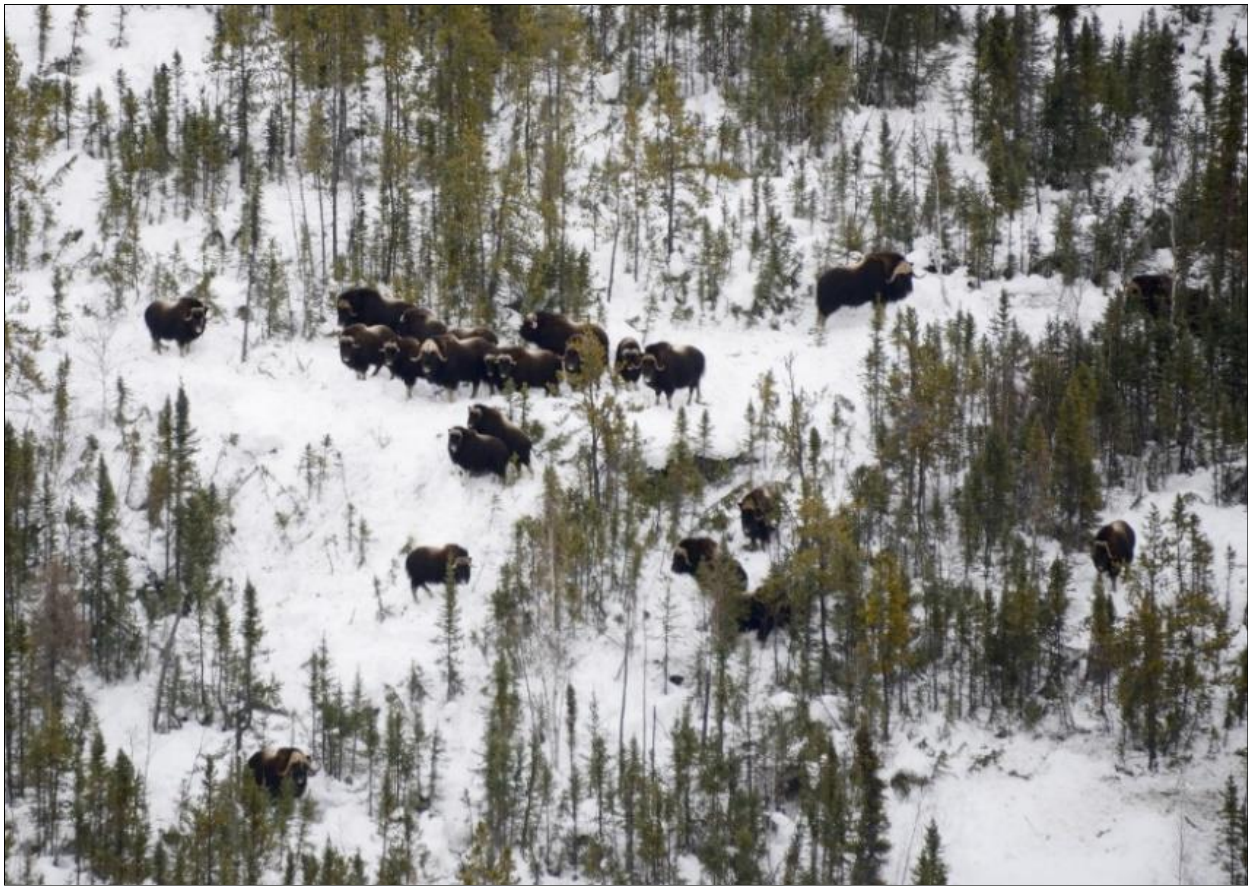
Figure S1 Muskox group containing many cows and juveniles observed in South Great Slave region Northwest Territories winter 2018. Photo Kristen Olesen.

Northwest Territories Arctic islands

For most NT Arctic islands, survey recurrence has been highly variable and usually infrequent. The exception is Banks Island, which since 1982 has been surveyed every two to four years for a total of 10 surveys over a 32-year period (1982 – 2014) (Latour 1985; McLean et al. 1986; McLean and Fraser 1992; Fraser et al. 1992; Nagy et al. 2006; Nagy et al. 2009a,b; Nagy et al. 2013a,b; Davison et al. 2013, 2017). Surveys using the fixed-wing strip-transect method have remained consistent throughout (Davison et al. 2017). Initially there was a large increase to ca 70,000 muskoxen (1994 and 2001). By 2010, however, this declined to ca 37,000, followed by the 2014 estimate of only ca 14,000 animals, indicating a rapid decline. Muskoxen on northwest Victoria Island, meanwhile, have apparently remained stable for about a decade. Muskoxen on the NT Arctic islands may total ca 32,284.

Nunavut mainland

Prior to 2013, NU muskox management boundaries were harvest based. This was changed to muskox management units that generally reflect actual muskox populations, based on genetics and consultations with traditional ecological knowledge holders (Leclerc in prep; Fig 2). Mainland NU muskox surveys, meanwhile, experienced the same problems as elsewhere. Given the additional complication of new management units, it reinforces the numerous impediments to establishing status and trends. Recently, muskox numbers have increased (substantially in the eastern mainland), expanding their distribution to re-colonize former habitats. Despite this expansion, there are no muskoxen present on the Melville Peninsula, even though that area is contained within the MX-10 boundaries. Similar to the

NT, muskoxen also expanded their distribution to an undetermined extent into the provinces below 60° N. Latitude. The exception is the Thelon Wildlife Sanctuary (MX-12), which was surveyed only once, 1994, and recent local knowledge indicates abundance is declining. Coincidentally, within MX-11 (Table 1, Fig. S2) muskox distribution changed to predominate in the northwest portion. The suggested driver was predator avoidance at the NT border. The population estimate for mainland NU is ca 27,296 muskoxen.

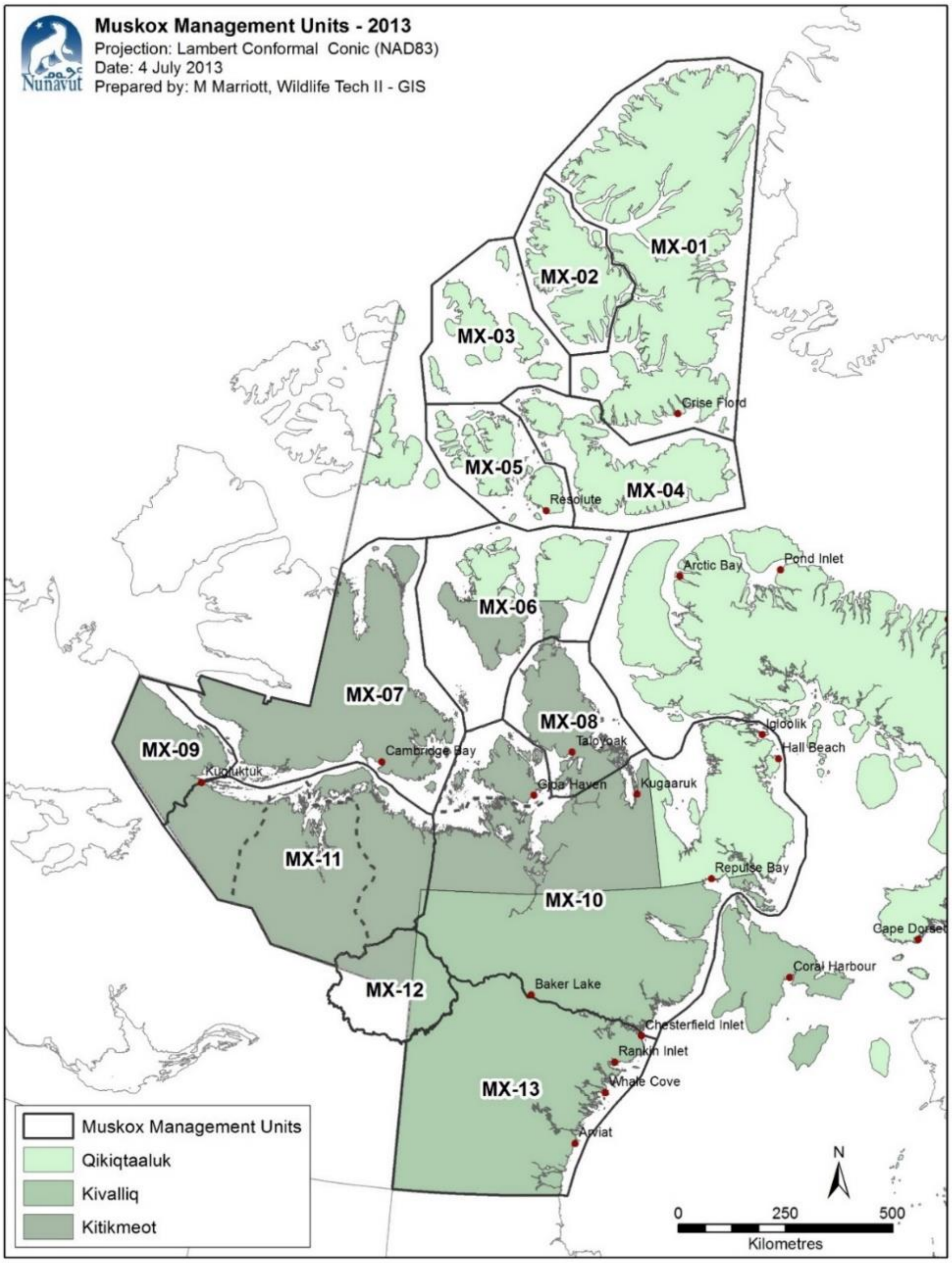

Figure S2 The Nunavut muskox management units as designated in 2013 (from Government of Nunavut Department of Environment. 2013).

Nunavut Arctic islands

For the Arctic islands of NU, surveys have also been infrequent, employing highly variable survey methods over inconsistent survey areas. Recently, substantial declines have been reported on eastern Victoria Island (MX-07). Abundance trend is uncertain on Prince of Wales and Somerset Islands (MX-06), but abundance has increased substantially since the early 2000's on Bathurst Island (MX-05), Devon Island (MX-04) and southern Ellesmere Island (MX-01). Northern Ellesmere Island (also MX-01), meanwhile, was estimated to have 8,115 muskoxen in 2006. The most recent population estimates for all NU islands indicate 32,502 muskoxen.

Quebec – Nunavik (Brodeur and Ford pers. comm.)

Although Quebec was never historical muskox range, the province now has an established population of the *O.m. wardi* subspecies. In 1967, 15 muskoxen captured near Eureka, Ellesmere Island, were taken to the community of Kuujjuaq, Ungava Bay, Quebec with the intention of establishing a muskox farm, provide local employment and build up a stock for introduction into northern Quebec (Chubbs and Brazil 2007). The qiviut wool portion of the project was unsuccessful, and from 1973 to 1983, the 54 captive muskoxen were gradually released at three nearby sites (Le Hénaff 1985). The now free-ranging population dispersed. Since their release, the distribution and abundance of the Nunavik muskox population has been estimated from sightings of mixed groups and partial surveys. Although considered a single population, the muskoxen occupy two distinct areas, Ungava Bay and East Hudson Bay, with the latter derived from the former. Exchanges occur between the two at an unknown frequency. In Nunavik, observations are most common along the Ungava Bay coast, between the communities of Kuujjuaq and Kangiqsujuaq, and on the East Hudson Bay coast, between Umiujaq and Inukjuak. Occasional sightings, typically of lone bulls, occur in areas as far south as Chisasibi and Schefferville (54°N). From 1988 to 2006, there were also scattered sightings of a few lone bulls in Labrador (Chubbs and Brazil 2007) but the species has not yet colonized the Ungava east of Kuujjuaq. Although never surveyed, in 2016 the 'Best Guess' estimate for the East Hudson Bay muskox population was 1,000 animals. This 'Best Guess' was also applied to the Ungava Bay population for 2016. In March 2019, the first systematic survey of the Ungava Bay population occurred, and provided a count of 3,000 muskoxen. Data analyses are expected to provide an estimate of abundance, with SE, CV and 95% CI's. Until then, the 2019 conservative estimated of the combined abundance for both Quebec populations is 4,000 muskoxen.

Greenland

For Greenland, muskox surveys have occurred infrequently or in some areas not at all. Endemic muskoxen, subspecies *O.m. wardi*, in Greenland traditionally inhabited the entire northwest, north and northeast of Greenland, and arrived by crossing the 20 km wide ice covered Nares Strait, which separates Greenland from Ellesmere Island, Canada (Bennike 1997, Bennike and Andreasen 2005). Northwest Greenland includes Thule/Qaanaaq (Inglefield Land, Mac Cormick Fiord, Cape Atholl), Washington Land and Hall Land. With the exception of Inglefield Land, muskoxen were extirpated in northwest Greenland within the last 100 years, and expedition hunting may have been a factor (Jensen 1929; Bennike 2002, 2013; Bennike and Andreasen 2005). In 1986, muskoxen were re-introduced to Inglefield Land, Mac Cormick Fiord, and Cape Atholl (details below). Further, in 2015, muskoxen were observed on Washington Land and Hall Land, but it is unknown if they represent a preexisting population or recent recolonization of these areas (Dalerum et al. 2017, 2018).

Historical (1800's) ship logbooks from whalers and expeditions to the northeast Greenland coast rarely observed muskoxen prior to 1870, suggesting low abundance before that time. By 1941, with knowledge from expeditions, resident trappers and harvest records, Jennov (1941) estimated 17,000

muskoxen, with the majority occurring in the Scoresbysund districts, including Jameson Land. In 1990, the 'Best Guess' population estimate for the entire Northeast Greenland (including Jameson Land) was 9,500 – 12,500 muskoxen (Boertmann et al. 1992). Similar to Jennov (1941), by the end of the 20th century, Jameson Land was considered to contain the majority of the muskoxen inhabiting Northeast Greenland (Hansen et al. 2012), and the Jameson Land sub-population may have peaked in abundance ca 1985 (Aastrup and Mosbech 2000). Distribution during the late 1900s indicated large numbers of muskoxen in the Colorado Dal sub-area of Jameson Land (Aastrup and Mosbech 2000). If abundance in the Colorado Dal sub-area of Jameson Land reflects overall population size, then the low numbers of muskoxen observed in 2008, 2009 and 2016 (Glahder et al. 2010; Boertmann and Nielsen 2010) suggest a possible declining abundance in the Jameson Land muskox population. Otherwise, overall trends for North East Greenland are unknown with the exception of one locality, Zackenberg (Fig 2), where muskox counts occur each summer. There, muskox abundance peaked a decade ago and declined rapidly thereafter (Schmidt et al. 2015). The need for reliable muskox monitoring covering the entire north and northeast was first noted by Jennov (1941). Eighty years later, monitoring and knowledge remain insufficient for management decisions.

Muskoxen were never endemic to the west or southwest coasts of Greenland, likely due to the heavily glaciated expanse of Melville Bay preventing southward dispersal from the Thule region (Bennike and Andreasen 2005). In the 1960s, 27 muskoxen, subspecies *O.m. wardi*, captured in Northeast Greenland were translocated to the Kangerlussuaq region (Søndre Strømfjord) in West Greenland (Vibe 1971). Abundance grew steadily over the first four decades. Minimum counts between 2000 and 2006 typically observed ca 4000-5000 individuals. Distance sampling based survey methods in 2018 estimated ca 20,000 muskoxen in the Kangerlussuaq region. Despite the 2018 estimate, in the areas accessible to hunters, local knowledge and minimum counts indicate a decline in muskox numbers since 2006.

Using animals captured from the successfully established Kangerlussuaq population, seven subsequent translocations occurred. These began in 1986 with three re-introductions to historical range in the Thule/Qaanaaq region of Northwest Greenland: Cape Atholl (n=7), Mac Cormick Fjord (n=6) and Inglefield Land (n=14). At that time, endemic muskoxen had disappeared from all but the latter, Inglefield Land, where an unknown number remained at the time of the re-introduction. In 1999, the estimated number of muskoxen in Inglefield Land was 270, while a 2017 minimum count of Cape Atholl observed 212 animals. Muskoxen were extirpated from Mac Cormick Fjord almost immediately. Current abundance may be ca 500 muskoxen in the Thule/Qaanaaq region of Northwest Greenland.

Following the Thule/Qaanaaq introductions, muskoxen captured from the Kangerlussuaq population were translocated to three more sites in Greenland, Ivittuut (1987, n=15), Svartenhuk (1991, n=31) and Naternaq (1993, n=31). The Ivittuut population currently numbers ca 800 – 1000 muskoxen. The recent decline of the Ivittuut population is entirely due to targeted harvest management aimed at reducing muskox density in this small ca 430 km^2 region. The Svartenhuk minimum count of 2002 observed 193 animals. The Naternaq minimum count in 2004 observed 112 muskoxen. In 2014, the latest translocation of muskoxen (n=19) was to Nanortalik, and took animals captured from Ivittuut. Calves were observed in spring 2017 and 2018. Translocating muskoxen to two further areas on the coast of West Greenland, i.e., Qeqertarsuaq Island and Paamiut, is under consideration in 2019. With the exception of the recent Nanortalik translocation and the protected muskoxen of the Northeast National Park, all other Greenland populations are under harvest regulation. Current estimate for total number of muskoxen in Greenland is ca 39,427, which is 23.4% of the global total.

Norway (Lønø 1960, 1962; Bretten pers. comm.)

Earlier (1932 and 1938) introductions to Norway of 10 and 2 calves respectively failed to establish populations (see below). The muskox population, subspecies *O.m. wardi*, currently established in the Dovre National Park region of Norway, is the result of several translocations, totaling 21 calves (out of a

total 27 brought to Norway) that were captured in East Greenland between 1947-1953. Initially disease and accidents claimed many of the translocated animals. By 1959, there remained only 14 muskoxen and poaching was considered a problem. Since then the population has grown. Although free ranging, the current population is managed intensively, e.g., animals leaving the park or posing a human safety risk are normally culled. Disease, collisions with trains, and culling are the major causes of mortality. Orf, also known as contagious ecthyma (*Parapoxvirus*), outbreaks occur generally every fourth year. Since 2004, the Dovre population has fluctuated between 170 and 300 muskoxen distributed over 250-280 km^2 in the Dovre National Park. Unsystematic snowmobile surveys cover 310 km^2. In March 2017, the minimum count was 249 animals, and the 2018 winter count was 244 muskoxen. The spring 2018 calf recruitment was 57 calves per 100 cows (age ≥ 3 years). For the future, management will regulate population size at ca 200 winter individuals.

Sweden (Bretten pers. comm.)

Despite Norwegian authorities actively culling any of their dispersing Dovre muskoxen, in 1971, five muskoxen successfully immigrated east across the border into Sweden. Present location is near or in the Rogen Nature Reserve, which is close to the Swedish-Norwegian border. Animals stray on both sides of the border, using both Fedmundsmarka, Røros, Norway and the Funäsdalen area in Sweden. By the mid-1980s, the Swedish population was 36 animals; however, thereafter numbers declined stabilizing at approximately seven individuals in the period 2005-2010 (Thulin et al. 2011). In 2017, there were 10 individuals, all of whom had names.

Russia

Endemic muskoxen disappeared about 2000 years ago. Beginning in 1974 muskoxen, subspecies *O.m. wardi*, were translocated to re-colonize the Russian mainland tundra on the Taimyr Peninsula, and in 1975 to Wrangel Island (Sipko and Gruzdev 2006). Taimyr received animals, 10 from Banks Island (NT, Canada) and 20 from Nunivak Island (Alaska, USA), while Wrangel Island's 20 animals came from Alaska's Nunivak Island (Vereshchagin et al. 2002, Sipko 2009). Since then, eight more regions of the Russian mainland tundra received muskoxen. Two further muskox populations (Putorana Plateau and Magadan Omulevka River), arose following natural expansion from established translocated populations. By early 2018, the result was 12 populations (Fig S3) (Taras Sipko pers. comm.). Today, several appear well established and most are growing in abundance (Taras Sipko pers. comm.). Details regarding Yakutia translocations may be found at http://dbr-yakutia.ru/in-english/muskox-resettlement/.

Discussion among Taras Sipko, Innokentiy Okhlopkov and Alexander Gruzdev designated the following four abbreviated names for translocated muskox populations now within the Sakha Republic, Yakutia, Russia:

1. Anabarskay (Анабарская): muskoxen on Anabar tundra, i.e., lower reaches of Anabar River.
2. Bulunskay (Булунская): muskoxen on Bulun tundra, i.e., Lena River delta/Haraulakskie Mountain.
3. Indigirskay (Индигирская): muskoxen on Indigir tundra, i.e., Chokurdah, lower reaches of Indigirka River/Kondakovskaâ Hill. Previously name Allaikhovsky (Алайховский РС (Якутия)).
4. Kolymshay (Колымская): muskoxen on Kolyma tundra, i.e., lower reaches of Kolyma River.

Translocations in Russia are ongoing, e.g., Bulunskay (Lena River Delta/ Haraulakskie Mountain) received 22 additional muskoxen in 2017, Yamal received 60 additional muskoxen in 2014-2016, and Chukotka has received multiple releases, the most recent being 2010 (Taras Sipko pers. comm.). Despite these, the Chukotka population remains small owing to illegal poaching and bear predation (Taras Sipko pers. comm.). Several further sites for muskox translocations are under consideration. One of these was achieved in summer 2018, when 25 juveniles were translocated to Zavyalova Island, which lies in the Pacific Ocean off the coast of the Magadan region (details in Table S3).

The muskox population on Wrangel Island is slowly increasing, although earlier thought stable at ca 800-900 animals and limited by the island's small area (Alexander Gruzdev pers. comm.). Monitoring abundance and composition of wild populations has become more frequent in recent years (Taras Sipko pers. comm.). Given recent estimates, Russian muskox abundance totals 15,796, which is 9.4% of the global total number of muskoxen. There is an increasing trend for most populations.

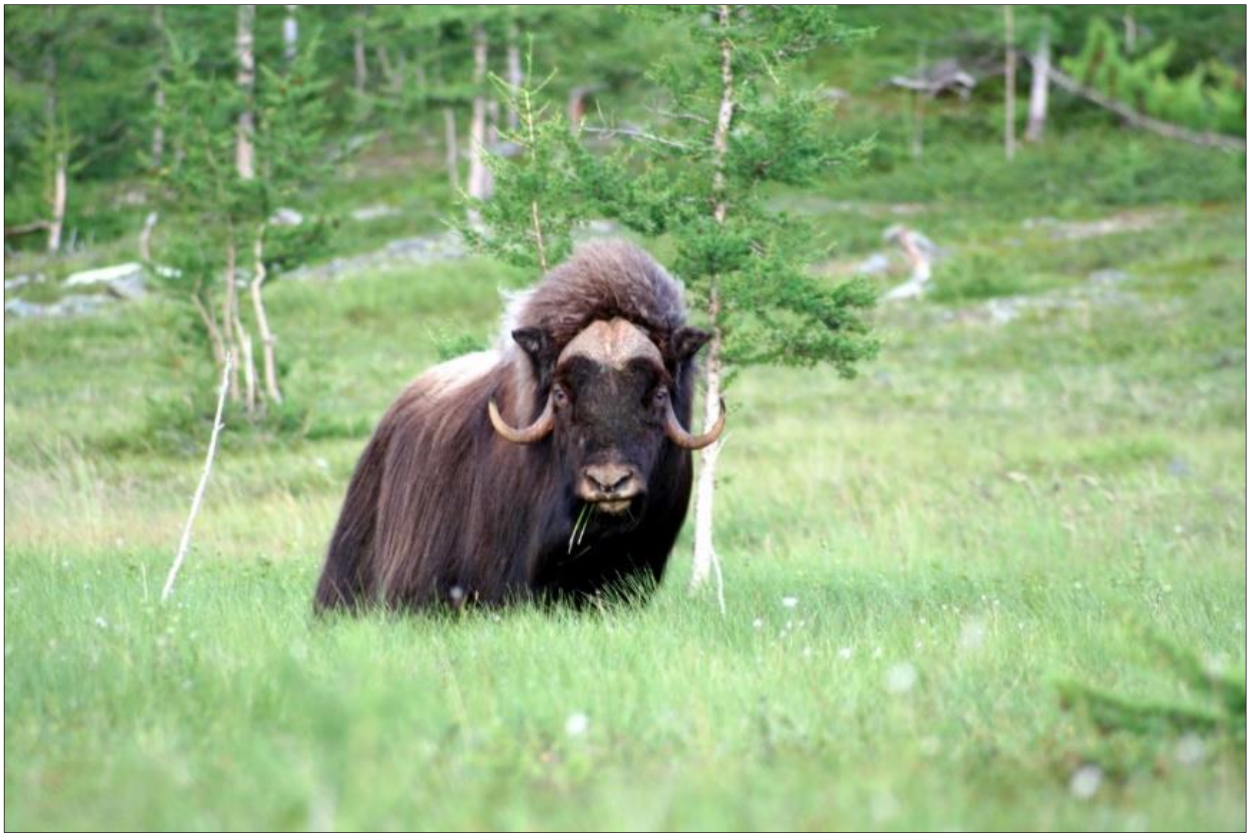

Figure S3 Mature muskox bull, summer 2014, standing in tall grass and taiga of Yamal region: Gorno Hadatinskij Natural Park (120 km from city of Salekhard). Photo Taras Sipko.

Captive Muskox Facilities

It is beyond the scope of this review to track the status of zoo muskox populations. The Copenhagen Zoo, Denmark, maintains a studbook with breeding records of captive muskox populations (Holst and Stelvig 2005).

In addition to zoos and educational game parks, captive research herds of muskoxen have come and gone. The University of Tromsø, Norway founded a small research herd ca. 1976 (Lent 1999), and one was established at the University of Saskatchewan (Flood et al. 1982). Although both are closed now, they provided a great deal of detailed information on nutrition, behavior, reproduction and disease in this species, much of which is incorporated into this review. Currently, the Robert G White Large Animal Research Station, operated by the University of Alaska Fairbanks, has continuously maintained a herd of muskoxen (*O.m. wardi*, originally captured on Nunivak Island in 1979) that over the years has fluctuated in size from 10-90 muskoxen. More recently, Russia has established captive facilities containing ca 150 muskoxen, among them the Yamal captive-breeding station (Taras Sipko pers. comm.).

These research facilities with captive research herds are valuable resources for the validation of techniques, technology and biological questions too difficult and/or expensive to control in the field.

Unsuccessful translocations

Most muskox translocations established new pockets of population, despite low founding numbers, and these increased in abundance and expanded their range. However, some translocations were unsuccessful and died out. Causes appear diverse and include too few translocated individuals, poaching, accidents and exposure of naïve-translocated juveniles to pathogens present in the new environment.

Greenland (Cuyler pers. comm.)

Seven muskoxen (*O.m. wardi*) re-introduced in 1986 to Mac Cormick Fjord in the Thule region of Northwest Greenland died before one year passed. Illegal harvest was the suspected cause, given close proximity of the Qaanaaq community.

Iceland (Lønø 1960)

In 1929, Iceland took seven muskoxen calves (*O.m. wardi*) from Northeast Greenland and released them at Gunnarsholt in south Iceland. Six calves died before the end of 1929, presumed owing to a sheep intestinal disease, Braxy (*Clostridium septicum*). The last calf died in 1931 bearing a heavy parasite load. In 1930, a further seven muskoxen calves, also from Northeast Greenland but via Norway, were brought to Iceland. Five were vaccinated and released at Gunnarsholt. All died by 1932. The remaining two calves, released near Grund Skorradal, West Iceland, also died by 1932.

Svalbard (Norway) (Lønø 1960)

Muskoxen were never endemic on Svalbard. In 1929, Norwegians captured 17 muskoxen calves (*O.m. wardi*) in Northeast Greenland, of which many overwintered first in Norway. The following year their new home became Nordaustlandet at Moskushamn in Advent fjord. For the first 3-4 years, all 17 thrived, and produced calves. They appeared to increase slowly or remain stable in abundance until 1941. During WWII, however, soldiers shot at least 19, and evacuating Russians released sled dogs, which killed some more. After the 1960's, numbers dwindled, calf mortalities occurred and the population was extirpated by 1983-85 (Grubb 2005). Early 1980s local knowledge among Norwegian miners alluded to illegal harvesting (Cuyler pers. comm.). Given that the distribution of the few remaining muskoxen (below sea cliffs at Grumantbyen / Colesdalen) was between two active mining towns poaching may have contributed to their demise.

Norway (Lønø 1960)

Norwegians translocated muskox calves (*O.m. wardi*) captured in Northeast Greenland to three places.

In 1924, Ålesund received 11 calves. Five died immediately. In 1925, they moved the remaining six to an island, Gurksøy, 35 km south of Ålesund. In 1926, two more muskoxen were released on Gurksøy. Although some calving occurred, the herd was gone ca 1927 and authorities cited poaching as the primary cause.

In 1932, Dovre received 10 calves, and authorities forbid all hunting. Although calving occurred, avalanches reduced numbers in 1933-34, and poaching was considered a problem. WWII exacerbated the poaching, and by 1945, nothing remained of this population.

In 1948, Bardufoss received 10 calves. Two escaped their enclosure and died. Calving was never observed, and although three animals remained in 1959, this translocation was not a success.

Sweden (Lønø 1960)

In 1900, the Swedes translocated four muskoxen calves (*O.m. wardi*) captured in Northeast Greenland to Medstugan in Jämtland, central Sweden. By 1902-03 all had died. In 1901, they released two muskoxen at Holmfors near Boden in northern Sweden; however, by 1903 these also had died.

REFERENCES (additional to those in manuscript)

Aastrup, P. and A. Mosbech. 2000. Population demography of the muskoxen in Jameson Land, 1982-1990. *Rangifer* 20(4): 229-238.

Adamczewski, J.Z, P.J. Fargey, B. Laarveld, A. Gunn, and P.F. Flood. 1998. The influence of fatness on the likelihood of early-winter pregnancy in muskoxen (*Ovibos moschatus*). *Theriogenology* 50(4): 605-614.

Adamczewski, J.Z, W.M. Kerr, E.F. Lammerding, and P.F. Flood. 1994. Digestion of low protein grass hay by muskoxen and cattle. *Journal of Wildlife Management* 58: 679-685.

Afema, J.A. 2008. Investigation of the role of diseases and trace minerals in the decline of Eastern North Slope Alaskan muskoxen (*Ovibos moschatus*). MPVM Thesis. Davis, California, USA: University of California.

Afema, J.A., K.B. Beckmen, S.M. Arthur, H.B. Huntington, and J.A. Mazet. 2017. Disease Complexity in a Declining Alaskan Muskox (*Ovibos moschatus*) Population. *Journal of Wildlife Disease* 53(2): 311-29.

Alaska Department of Fish and Game. 2017. The status of muskox and factors influencing their populations. Division of Wildlife Conservation, Annual Performance Report 1 July 2016-30 June 2017, Federal Aid in Wildlife Restoration Project 16.0, Juneau.

Alendal, E. 1971. Early sexual maturity and high fecundity in wild muskoxen in Norway. *Fauna* 24: 96-100.

Alendal, E., and O. Helle. 1983. Helminth parasites of muskoxen (*Ovibos moschatus*) in Norway incl. Spitsbergen and in Sweden, with a synopsis of parasites reported from this host. *Fauna Norvegica Series A* 4: 41-52.

Barboza, P.S., and J.E. Blake. 2001. Ceruloplasmin as an indicator of copper reserves in wild ruminants at high latitudes. *Journal of Wildlife Disease* 37(2): 324-331.

Barboza, P.S., E.P. Rombach, J.E. Blake, and J.A. Nagy. 2003. Copper status of muskoxen: a comparison of wild and captive populations. *Journal of Wildlife Disease* 39(3): 610-619.

Barr, W. 1991. Back from the Brink: The road to muskox conservation in the Northwest Territories. Arctic Institute of North America, Komatik Series No. 3, Calgary, Alberta, Canada.

Bennike, O. 1997. Quaternary vertebrates from Greenland: a review. *Quaternary Science Reviews* 16: 899–909.

Bennike, O. 2002. Late Quaternary history of Washington Land, north Greenland. *Boreas* 31: 260–272.

Bennike, O. 2013. Radiocarbon dating of musk-ox (*Ovibos moschatus*) bones from the Thule region, northwest Greenland. *Polar Record* 50. 113-118. 10.1017/S0032247413000193.

Bennike, O., and C. Andreasen. 2005. New dates of muskox (*Ovibos moschatus*) remains from northwest Greenland. *Polar Record* 41: 125–129.

Berger, L., R. Speare, P. Daszak, D.E. Green, A.A. Cunningham, C.L. Goggin, R. Slocombe, M.A. Ragan, A.D. Hyatt, and K.R. McDonald. 1998. Chytridiomycosis causes amphibian mortality associated with population declines in the rain forests of Australia and Central America. *Proceedings of the National Academy of Sciences* 95: 9031–9036.

Blake, J.E., and B.D. McLean. 1988. Epidemiology of yersiniosis in muskoxen on Banks Island, NWT.

Proceedings for the joint conference of the American Association of Zoo Veterinarians and the American Association of Wildlife Veterinarians, Toronto, Ontario, Canada, 6-10 November 1988. Philadelphia: American Association of Zoo Veterinarians; p. 71-72.

Blake, J.E., B.D. McLean, and A. Gunn. 1991. Yersiniosis in free-ranging muskoxen on Banks Island, Northwest Territories, Canada. *Journal of Wildlife Disease* 27(4): 527-33.

Blakley, B.R., S.J. Kutz, S.C. Tedesco, and P.F. Flood. 2000. Trace mineral and vitamin concentrations in the liver and serum of wild muskoxen from Victoria Island. *Journal of Wildlife Disease* 36(2): 301-7.

Blehert, D.S., A.C. Hicks, M. Behr, C.U. Meteyer, B.M. Berlowski-Zier, E.L. Buckles, J.T. Coleman, S.R. Darling, A. Gargas, R. Niver, J.C. Okoniewski, R.J. Rudd, and W.B. Stone. 2009. Bat white-nose syndrome: an emerging fungal pathogen? *Science* 323: 227–227. doi: 10.1126/science.1163874.

Boertmann, D., M.C. Forchhammer, C.R. Olesen, P. Aastrup, and H. Thing. 1992. The Greenland muskox population status 1990. *Rangifer* 12(1): 5-12.

Boertmann, D. & Nielsen, R.D. 2010. Geese, seabirds and mammals in North and Northeast Greenland. Aerial surveys in summer 2009. National Environmental Research Institute, Aarhus University. 66 pp. – NERI Technical Report No. 773.

Campos, P. F., E. Willerslev, A. Sher, L. Orlando, E. Axelsson, A. Tikhonov, K. Aaris-Sørensen, A.D. Greenwood, et al.. 2010. Ancient DNA analyses exclude humans as the driving force behind late Pleistocene musk ox (*Ovibos moschatus*) population dynamics. *Proceedings of the National Academy of Sciences* 107(12): 5675-5680.

Chubbs, T.E., and J. Brazil. 2007. The occurrence of muskoxen, *Ovibos moschatus* in Labrador. *Canadian Field-Naturalist* 121(1): 81-84.

Dalerum, F., L. Dalén, C. Fröjd, N. Lecomte, Å. Lindgren, T. Meijer, P. Pečnerová, and A. Angerbjörn. 2017. Spatial variation in Arctic hare (*Lepus arcticus*) populations around the Hall Basin. *Polar Biology* doi: 10.1007/s00300-017-2116-1

Dalerum, F., S. Freire, A. Angerbjörn, N. Lecomte, Å. Lindgren, T. Meijer, P. Pečnerová, L. Dalén. 2018. Exploring the diet of arctic wolves (*Canis lupus arctos*) at their northern range limit. *Canadian Journal of Zoology* 96(3): 277–281. doi:10.1139/cjz-2017-0054

Das Neves, C., K. Handeland, T. Øren, K. Madslien, and T. Bretten. 2016. Systematic Health Monitoring of the Norwegian Muskox Population: 12 years of surveillance and interventions. In: 1st Muskox Health Ecology Symposium 2016, 7-10 November, Calgary Alberta Canada – Abstract book page 28.

Davidson, R.K, H. Amundsen, L. N. Oftenes, K. Luyckx, L.J Robertson, G.G. Verocai, S.J. Kutz, and B. Ytrehus. 2014. Sentinels in a climatic outpost: endoparasites in the introduced muskox (*Ovibos moschatus wardi*) population of Dovrefjell, Norway. *International Journal for Parasitology: Parasites and Wildlife* 3(2): 154-160.

Davison, T., J. Pongracz, and J. Williams. 2013. Population survey of Peary caribou (*Rangifer tarandus pearyi*) and muskoxen (*Ovibos moschatus*) on Banks Island, Northwest Territories, July 2010. *Rangifer* 33, Special Issue No.21: 135-140.

Davison, T., J. Williams, and J. Adamczewski. 2017. Aerial Survey of Peary Caribou (Rangifer tarandus pearyi) and Muskoxen (Ovibos moschatus) on Banks Island, July 2014. Department of Environment and Natural Resources, Government of the Northwest Territories. Manuscript Report No 270: 25 pp.

Desforges, J.P., L. Jaspersen, T.H. Jensen, C. Grøndahl, M.F. Bertelsen, S.D. Guise, C. Sonne, R. Dietz, et al. 2018. Immune function in arctic mammals: Natural killer (NK) cell-like activity in polar bear, muskox and reindeer. *Veterinary Immunology and Immunopathology* 195: 72-75.

Dieterich, R.A, G.R. Spencer, D. Burger, A.M. Gallina, J. VanderSchalie. 1981. Contagious Ecthyma in Alaskan muskoxen and Dall sheep. *Journal of American Veterinary Medicine Association* 179: 1140-1143.

Falk, E.S. 1978. Parapoxvirus infections of reindeer and muskox associated with unusual human

infections. *British Journal of Dermatology* 99: 647-654.
Flood, P., J.E. Rowell, G.J. Glover, R.K. Chaplin and S. Humphreys. 1984. The establishment of a small herd of captive muskoxen. In D.R. Klein, R.G. White, and S. Keller, eds. Proceedings of the First International Muskox Symposium. Biol. Pap. Univ. Alaska Spec. Rep. No. 4
Forbes, L.B. 1991. Isolates of *Brucella suis* biovar 4 from animals and humans in Canada, 1982-1990. *The Canadian Veterinary Journal* 32(11): 686-688.
Fraser, P., A. Gunn, and B. McLean. 1992. Abundance and distribution of Peary Caribou and Muskoxen on Banks Island, NWT, June 1991. Renewable Resources, Government of the Northwest Territories. Manuscript Report No. 63. 18pp.
Gates, C.C., G. Wobeser, and L.B. Forbes. 1984. Rangiferine brucellosis in a Muskox, *Ovibos moschatus* moschatus (Zimmermann). *Journal of Wildlife Disease* 20(3): 233-234.
Glahder, C.M., D. Boertmann, J. Madsen, M. Tamstorf, K. Johansen, J. Hansen, A. Walsh, C. Jaspers, and M. Bjerrum. 2010. Biological baseline study in the Ramsar site "Heden" and the entire Jameson Land, East Greenland. National Environmental Research Institute, Aarhus University. 86 p. – NERI Technical Report No. 769.
Gordeeva, N.V., T. Sipko, and A.R. Gruzdev. 2009. Microsatellite DNA variability in the populations of muskoxen *Ovibos moschatus* transplanted into the Russian north. *Russian Journal of Animal Genetics* 45: 932-940.
Government of Nunavut Department of Environment. 2013. *Management plan for High Arctic muskoxen of the Qikiqtaaluk region 2013 - 2018.* Iglulik, NU. 20pp.
Gray, D.R. 1987. The muskoxen of Polar Bear Pass. Fitzhenry and Whiteside, Markham, Ontario, Canada.
Grayson, D.K., and D.J. Meltzer. 2002. Clovis hunting and large mammal extinction: a critical review of the evidence. *Journal of World Prehist*ory 16: 313-359.
Grenfell, T.C., and J. Putkonen. 2008. A method for the detection of the severe rain-on-snow event on Banks Island, October 2003, using passive microwave remote sensing. *Water Resource Research*, 44: 3425-3434
Grubb, P. 2005. Order Artiodactyla. *In. Mammal Species of the World*, 3rd Edition, ed. D.E. Wilson, and D.M. Reeder, 637-722.The Johns Hopkins University Press, Baltimore.
Guthrie, R.D. 2001. Origin and causes of the mammoth steppe: a story of cloud cover, woolly mammoth tooth pits, buckles and inside-out Beringia. *Quaternary Sci. Rev*. 20: 549-574.
Guthrie, R.D. 2006. New carbon dates link climatic change with human colonization and Pleistocene extinctions. *Nature* 441: 207-209.
Handeland, K., L. Z. Muzs, C. Cuyler, M. Heum, F. Suhel, and C. G. das Neves. 2018. Gammaherpesvirus (Type 1 Ruminant Rhadinovirus) in Muskox (*Ovibos moschatus*) in Greenland. Journal of Wildlife Diseases 54: 400-402.
Handeland, K., T. Tengs, B. Kokotovic, T. Vikøren, R.D. Ayling, B. Bergsjø, O.G. Sigurðardóttir, and T. Bretten. 2014. Mycoplasma ovipneumoniae--a primary cause of severe pneumonia epizootics in the Norwegian Muskox (*Ovibos moschatus*) population. *PLoS One* 9(9): e106116.
Hansen, J., G. Asmund, P. Aastrup, A. Mosbech, C. Bay, M. Tamstorf, and D. Boertmann. 2012. Jameson Land: a strategic environmental impact assessment of hydrocarbon and mining activities. Danish Centre for Environment and Energy Report No. 41. http://www.dmu.dk/Pub/SR41.pdf
Hoberg, E.P., L. Polley, A. Gunn, and J.S. Nishi. 1995. *Umingmakstrongylus pallikuukensis* gen. nov. et sp. nov. (Nematoda: Protostrongylidae) from muskoxen*, Ovibos moschatus*, in the central Canadian Arctic, with comments on biology and biogeography. *Canadian Journal of Zoology* 73(12): 2266-2282.
Hoberg, E.P., S.J. Kutz, J. Nagy, E. Jenkins, B. Elkin, M. Branigan, and D. Cooley. 2002. Protostrongylus stilesi (Nematoda: Protostrongylidae): ecological isolation and putative host-switching between Dall's sheep and muskoxen in a contact zone. *Comparative Parasitology* 69: 1–9.

Hoberg, E.P., K.J. Monsen, S. Kutz, and M.S. Blouin. 1999. Structure, biodiversity, and historical biogeography of nematode faunas of holarctic ruminants: morphological and molecular diagnoses for Teladorsagia boreoarcticus n. sp. (Nematoda: Ostertagiinae), a dimorphic cryptic species in muskoxen, *Ovibos moschatus*. *Journal of Parasitology* 85: 910–934.

Holst, B., and M. Stelvig. 2005. International Studbook for the Muskox (Ovibos moschatus). Volume III. Copenhagen Zoo, Roskildevej 38, DK-2000 Frederiksberg, Copenhagen, Denmark. 91 pp.

IUCN 2018. IUCN Red List of Threatened Species. Version 3.1 www.iucnredlist.org

Jenkins, D., M. Campbell, G. Hope, J. Goorts, and P. McLoughlin. 2011. Recent trends in abundance of Peary Caribou (*Rangifer tarandus pearyi*) and muskoxen (*Ovibos moschatus*) in the Canadian Arctic Archipelago, Nunavut. Department of Environment, Government of Nunavut, Wildlife Report 1, Pond Inlet, Canada.

Jensen, S. 1929. Moskusoksen paa Grønland og dens fremtid. *In* the Report of the 18. Scandinavian Naturalist Congress in Copenhagen, 26-31 August 1929. Royal Danish Library, Polar Library, Copenhagen, Denmark. 8 pp. (in Danish)

Department of Fish and Game, Species Management Report ADF&G/DWC/SMR-2015-2, Juneau

Jingfors, K.T., and D.R. Klein. 1982. Productivity in recently-established muskox populations in Alaska. *Journal of Wildlife Management* 46: 1092-1096.

Jørgensen, T., T. Traavik,S.D. Mathiesen, and A.S. Blix. 1984. On contagious ecthyma and its treatment in muskoxen. In: ed. D.R. Klein, R.G. White, and S. Keller. Proceedings of the First International Muskox Symposium. Fairbanks: *Biological Papers University Alaska Special Report* 4: 182.

Kafle, P., J. Sullivan, G.G. Verocai, and S.J. Kutz. 2017. Experimental Life-Cycle of *Varestrongylus eleguneniensis* (Nematoda: Protostrongylidae) in a Captive Reindeer (*Rangifer tarandus tarandus*) and a Muskox (*Ovibos moschatus moschatus*). *Journal of Parasitology* 103(5): 584-587.

Klein, D.R., and C. Bay. 1990. Foraging dynamics of muskoxen in Peary Land, northern Greenland. *Ecography* 13(4): 269-280.

Klein, D.R., and C. Bay. 1991. Diet selection by vertebrate herbivores in the High of Greenland. *Holarctic Ecology* 14: 152-155.

Kock, R.A., M. Orynbayev, S. Robinson, S. Zuther, N.J. Singh, W. Beauvais, E.R. Morgan, A. Kerimbayev, S. Khomenko, and H.M. Martineau. 2018. Saigas on the brink: Multidisciplinary analysis of the factors influencing mass mortality events. *Science advances* 4: eaao2314.

Korsholm, H., and C.R. Olesen. 1993. Preliminary investigations on the parasite burden and distribution of endoparasite species of muskox (*Ovibos moschatus*) and caribou (*Rangifer tarandus groenlandicus*) in West Greenland. *Rangifer* 13(4): 185-189.

Kristensen, D.K., E. Kristensen, M.C. Forchhammer, A. Michelsen, and N.M. Schmidt. 2011. Arctic herbivore diet can be inferred from stable carbon and nitrogen isotopes in C_3 plants, faeces and wool. *Canadian Journal of Zoology* 89: 892-899.

Kummeneje, K., and J. Krogsrud. 1978. Contagious ecthyma (Orf) in the muskox (*Ovibos moschatus*). *Acta Veterinaria Scandinavica* 19: 461-462.

Kutz, S.J., J. Ducrocq, G.G. Verocai, B.M. Hoar, D.D. Colwell, K.B. Beckmen, L. Polley, B.T. Elkin et al. 2012. Parasites in ungulates of Arctic North America and Greenland: a view of contemporary diversity, ecology, and impact in a world under change. 99–252. Elsevier. Advances in Parasitology.

Kutz, S.J., E.P. Hoberg, J. Nagy, I. Polley, and B. Elkin. 2004. “Emerging” parasitic infections in arctic ungulates. *Integrative and Comparative Biology* 44(2): 104-118.

Kutz, S.J. 1999. The biology of *Umingmakstrongylus pallikuukensis*, a lung nematode of muskoxen in the Canadian arctic: field and laboratory studies. PhD Thesis. Saskatoon, Saskatchewan, Canada: University of Saskatchewan.

Kutz, S.J., K. Fisher, and L. Polley. 1999a. A lung nematode in Canadian Arctic muskoxen. Standard radiographic and computed tomographic imaging. *Veterinary Clinics of North America: Food Animal Practice* 15: 359-377.

Kutz, S. J., E.P. Hoberg, and L. Polley. 1999b. Experimental infections of muskoxen (*Ovibos moschatus*) and domestic sheep with *Umingmakstrongylus pallikuukensis* (Nematoda: Postostonglydae): parasite development, population structure, and pathology. *Canadian Journal of Zoology* 77: 1562.

Lair, S., A. Santamaria-Bouvier, and M. Simard. 2016. Infections of muskoxen (*Ovibos moschatus*) from Nunavik by the giant liver fluke (*Fascioloides magna*). *In* Muskox Health Ecology Symposium Abstracts, Muskox Health 2016, Session 4. XXV, pp. 20-21.

Landa, A., S.R. Jeremiassen, and R. Anderson. 2000. Rensdyr of moskusokser I Inglefield Land, Nordvestgrønland. Teknisk rapport nr. 31. Pinngortitaleriffik, Grønlands Naturinstitut, Nuuk, Greeland. (in Danish).

Larter, N.C. 1999. An aerial survey of muskoxen in the Paulatuk area of the Inuvialuit Settlement Region, March 1997. Department of Resources, Wildlife, and Economic Development, Government of the Northwest Territories, Inuvik, NWT. Manuscript Report No. 116. 27pp

Latour, P.B. 1985. Population estimates for Peary caribou and muskoxen on Banks Island in 1982. NWT Wildlife Services, Government of the Northwest Territories. File Report No. 49. 21pp. Reanalyzed: Nagy J. A., P. Latour and W. H. Wright. 2009. Population Estimates for Peary Caribou and Muskox

Latour, P.B. 1987. Observations on demography, reproduction, and morphology of muskoxen (*Ovibos moschatus*) on Banks Island, Northwest Territories. *Canadian Journal of Zoology* 65: 265-269.

Le Hénaf, D. 1985. Introduction du boeuf musqué, *Ovibos moschatus*, au Nouveau-Québec et état actuel des populations en liberté. *Canadian Field-Naturalist* 99(1): 103-104. (in French)

Lenart, E.A. 2015. Units 26B and 26C muskox. Chapter 4, pages 1-26, in P. Harper and L.A. McCarthy, editors. Muskox management report of survey and inventory activities 1 July 2012 - 30 June 2014. Alaska Department of Fish and Game, Species Management Report ADF&G/DWC/SMR-2015-2, Juneau, Alaska, USA

Li, H., K. Gailbreath, L.C. Bender, K. West, J. Keller, T.B. Crawford. 2003. Evidence of three new members of malignant catarrhal fever virus group in muskox (*Ovibos moschatus*), Nubian ibex (*Capra nubiana*), and gemsbok (*Oryx gazella*). *Journal of Wildlife Disease* 39(4): 875-880.

Lønø, O. 1960. I. Transplantation of the muskox in Europe and North-America II. Transplantation of hares to Svalbard. Norsk Polarinstituttt. Meddelelser nr. 84. Universitetsforlaget Oslo.

Lønø, O. 1962. Aktuelt om moskusoksen. *Fauna* 15: 204-210. (In Norwegian)

Mathiesen, S.D., T. Jørgensen, T. Traavik, and A.S. Blix. 1985. On contagious ecthyma and its treatment in muskoxen. *Acta Veterinaria Scandinavica* 26: 120-126.

McLean, B. 1996. Yersiniosis as a mortality factor of muskoxen on Banks Island. Northwest Territories, Canada Fairbanks, AK, USA: MS thesis, University of Alaska Fairbanks.

Mclean, B.D., and F. Fraser. 1992. Abundance and distribution of Peary Caribou and muskoxen on Banks Island, NWT, June 1989. Renewable resources, Government of the Northwest Territories. File Report No. 106. 28pp.

McLean, B.D., P. Fraser, and J.E. Blake. 1993. Yersiniosis in muskoxen on Banks Island, NWT, 1987-1990. *Rangifer* 13(1): 65-66.

Mclean, B., K. Jingfors, and R. Case. 1986. Abundance and Distribution of Muskoxen and Caribou on Banks Island, July 1982. Renewable resources, Government of the Northwest Territories. File Report No. 64. 45pp.

Mulders, R. and M. Bradley. 1991. Distribution and abundance of muskoxen in Central Keewatin, NWT. DRWED. Arviat NT. Government of the NWT file report No.134. 32pp.

Nagy, J.A., A. Gunn, and W.H. Wright. 2009a. Population estimates for Peary caribou and muskox on Banks Island, NWT, August 1992. Environment and Natural Resources, Government of the

Northwest Territories. Manuscript Report No. 198. 41pp.
Nagy, J.A., A. Gunn, and W.H. Wright. 2009b. Population estimates for Peary caribou and muskox on Banks Island, NWT, July 2005. Environment and Natural Resources, Government of the Northwest Territories. Manuscript Report No. 200. 47pp.
Nagy, J.A., N. Larter, and W.H. Wright. 2006. Population estimates for Peary caribou and muskox on Banks Island, NT, July 2001. Environment and Natural Resources, Government of the Northwest Territories. Manuscript Report No. 199. 47pp.
Nagy, J.A., N. Larter, and W.H. Wright. 2013a. Population estimates for Peary caribou and muskox on Banks Island, NWT, July 1994. Environment and Natural Resources, Government of the Northwest Territories. Manuscript Report No. 223. 50pp.
Nagy, J.A., N. Larter, and W.H. Wright. 2013b. Population estimates for Peary caribou and muskox on Banks Island, NWT, July 1998. Environment and Natural Resources, Government of the Northwest Territories. Manuscript Report No. 224. 48pp.
Olesen, C.R. 1993. Rapid population increase in an introduced muskox population, West Greenland. *Rangifer* 13(1): 27-32.
Ott, J.T. 1998. Parasitic helminths of muskoxen (*Ovibos moschatus*) from Banks Island, Northwest Territories, Canada. MS Thesis. Fairbanks Alaska USA: University of Alaska Fairbanks.
Pedersen, A.B., K.E. Jones, C.L. Nunn, and S. Altizer. 2007. Infectious diseases and extinction risk in wild mammals*. Conservation Biology* 21: 1269–1279.
Pielou, E.C. 1991. After the Ice Age. The Return of Life to Glaciated North America. University of Chicago Press, Chicago.
Preece, N.D., S.E. Abell, L. Grogan, A. Wayne, L.F. Skerratt, P. van Oosterzee, A.L. Shima, P. Daszak, H. Field, A. Reiss, L. Berger, T.L. Rymer, D.O. Fisher, M.J. Lawes, S.G. Laurance, H. McCallum, C. Esson, and J.H. Epstein. 2017. A guide for ecologists: Detecting the role of disease in faunal declines and managing population recovery. *Biological Conservation* 214: 136–146. doi:10.1016/j.biocon.2017.08.014.
Raillard, M. 1992. Influence of muskox grazing on plant communities of Sverdrup pass (79˚N), Ellesmere Island, N.W.T., Canada. PhD Thesis. University of Toronto.
Rennert, K.J., G. Roe, J. Putkonen, and C.M. Bitz. 2009. Soil thermal and ecological impacts of rain on snow events in the circumpolar Arctic. American Meteorological Society. *Journal of Climate* 22: 2302-2315.
Reynolds, P.E. 1993. Dynamics of muskox groups in northeastern Alaska. *Rangifer,* 13(2)83-88.
Reynolds, P.E. 2001. Reproductive patterns of female muskoxen in northeastern Alaska. *Alces* 37(2): 403-401.
Reynolds, P.E. 2006. Muskoxen in Arctic National Wildlife Refuge Game Management Unit 26C 2005-2006. U.S. Fish and Wildlife Service, Arctic National Wildlife Refuge report, Fairbanks, Alaska. 16 pp.
Reynolds, P., E. Lenart, D. Cooley, I. McDonald, G. Carrol. 2011. Changes in distribution and abundance of muskoxen in Arctic National Wildlife Refuge and adjacent areas in Alaska and Yukon, Canada. Poster. Arctic Ungulate Conference. Yellowknife, Canada
Rombach, E.P. 2001. Trace mineral reserves for reproduction and development in muskoxen. MS Thesis. Fairbanks Alaska USA: University of Alaska Fairbanks.
Rombach, E.P., P.S. Barboza, and J.E. Blake. 2003. Costs of gestation in an Arctic ruminant: copper reserves in muskoxen. *Comparative Biochemical Physiology C-Toxicology and Pharmacology* 134(1): 157-168.
Rosa, C., D. Miller, M. J. Gray, A. Merrill, T. Vann, and J. Blake. 2007. Exploring passive transfer in muskoxen (*Ovibos moschatus*). *Journal Zoo Wildlife Medicine* 38: 55-61.
Rowell, J.E. 1991. Reproductive biology and endocrinology in captive muskoxen (*Ovibos moschatus*). PhD Thesis. Saskatoon, Canada: University of Saskatchewan.

Rowell, J.E., and P.F. Flood. 1988. Progesterone, oestadiol-17 and LH during the oestrus cycle of muskoxen (*Ovibos moschatus*). *Journal of Reproduction and Fertility* 84: 117-122.
Rowell, J.E., M.C. Sousa, and M.P. Shipka. 2007. Estrous synchronization in captive muskoxen: progesterone and the bull effect. *Canadian Journal of Animal Science* 87: 535-538.
Rowell, J.E., R.G. White, and W.E. Hauer. 1997. Progesterone during the breeding season and pregnancy in female muskoxen on different dietary regimens. *Rangifer* 17: 125-129.
Rozenfeld, S.B., A.R. Gruzdev, T.P. Sipko, and A.N. Tikhonov. 2012. Trophic Relationships of muskoxen (*Ovibos moschatus*) and reindeer (*Rangifer tarandus*) on Wrangel Island. *Biology Bulletin* 39(9): 779-787.
Samuel, W.M., and D.R. Gray. 1974. Parasitic infection in muskoxen. *The Journal of Wildlife Management* 38(4): 775–782.
Schaefer, J.A., and F. Messier. 1995a. Habitat selection as a hierarchy: the spatial scales of winter foraging by muskoxen. *Ecography* 18(4): 333–344.
Schaefer, J.A., and F. Messier. 1995b. Winter foraging by muskoxen: a hierarchical approach to patch residence time and cratering behavior. *Oecologia*. 104(1): 39-44.
Schaefer, J.A., and F. Messier. 1996. Winter activity of muskoxen in relation to foraging condition. *Ecoscience* 3: 147-153.
Schmidt, J.H., T.S. Gorn, and C. Westing. 2010. Using distance sampling to estimate muskoxern abuncance of the Seward Peninsula, Alaska. Prepared for the 2010 Sweard Peninsula Muskox Census by National Park Service and Alaska Department of Fish and Game.
Schmidt, N.M., T.B. Berg, M.C. Forchhammer, D.K. Hendrichsen, L.A. Kyhn, H. Meltofte,and T.T Høye. 2008. Vertebrate Predator--Prey Interactions in a Seasonal Environment. *Advances in Ecological Research* 40: 345-370.
Schmidt, N.M., S.H. Pedersen, J.B. Mosbacher, and L.H. Hansen. 2015. Long-term patterns of muskox (*Ovibos moschatus*) demographics in high arctic Greenland. *Polar Biology* 38(10): 1667-1675.
Shapiro, B., A.J Drummond, A. Rambaut, M.C. Wilson, P.E. Matheus, A.V. Sher, G.Oliver, M. Pybus2, et al. 2004. Rise and fall of the Beringian steppe bison. *Science* 306: 1561-1565.
Simpson, G.G. 1945. The principals of classification and a classification of mammals. *Bulletin of the American Museum of Natural History* 85: 1-350.
Sipko, T.P., and A.R. Gruzdev. 2006. Re-introduction of muskoxen in Northern Russia. *Re-introduction news IUCN/SSC* 25: 25-26.
Sipko, T.P. 2009. Status of reintroductions of three large herbivores in Russia. *Alces* 45: 23-29.
Smith, K.F., D.F. Sax, and K.D Lafferty. 2006. Evidence for the role of infectious disease in species extinction and endangerment. *Conservation biology* 20: 1349–1357.
Swor, R.M. 2002. The effect of a marginal copper diet on muskox calf growth and immune function. MS Thesis. Fairbanks Alaska USA: University of Fairbanks Alaska.
Tener, J.S. 1954. Facts about Canadian musk-oxen*. Transactions of the North American Wildlife Conference, Chicago, 1954*. Washington, D.C.: American Wildlife Institute pp. 504-510.
Tener, J.S. 1965. *Muskoxen in Canada: a biological and taxonomic review*. Canadian Wildlife Service Ottawa, Canada: Queen’s Printer. Monogram 2: 1-166.
Tessaro, S., J.E. Rowell, R. Cawthorn, and P. Latour. 1984. Banks Island Muskox Harvest, 1982. Pages 177-180 *in* D. R. Klein, R. G. White, and S. Keller, editors. First International Muskox Symposium. Bioklogical Papers of the University of Alaska, Fairbanks.
Thulin, C.G., L. Englund, G. Ericsson, and G. Spong. 2011. The impact of founder events and introductions on genetic variation in the muskox *Ovibos moschatus* in Sweden. *Acta Theriologica* 56: 305-314.
Tomaselli, M. 2018. Improved Wildlife Health and Disease Surveillance through the Combined Use of Local Knowledge and Scientific Knowledge. PhD Thesis, Faculty of Veterinary Medicine, Calgary AB, Canada: University of Calgary. http://hdl.handle.net/1880/107597

Tomaselli, M., C. Dalton, P.J. Duignan, S. Kutz, F. van der Meer, P. Kafle, and S. Checkley. 2016. Contagious Ecthyma, Rangiferine Brucellosis, and Lungworm Infection in a Muskox (*Ovibos moschatus*) from the Canadian Arctic, 2014. *Journal of Wildlife Diseases 52*(3): 719-724.

Tryland, M., K.B. Beckmen, K.A. Burek-Huntington, E.M. Breines, and J. Klein. 2018. Orf virus infection in Alaskan mountain goats, Dall's sheep, muskoxen, caribou and Sitka black-tailed deer. *Acta Veterinaria Scandinavica* 60(1): 12.

Vereshchagin, N., V. Zabrodin, Y. Karbainov, N. Lovelius, and V. Tikhonov. 2002. The muskox in the tundra of Russia. Experiment of the XX century in the renewal of lost species. Editor. Dr.Sci.Biol. Prof., Academician of the Petrovskaya academy of sciences and arts N. Lovelius - Saint Petersburg, Asterion. ISBN 5-94856-009-0.

Vibe, C. 1971. Moskusoksen. –In: A. Nørrevang, T.J. Meyer, and S. Christensen (eds.). *Danmarks natur, Grønland og Færoerne*. Politikens Forlag. Pages 401-404. (in Danish).

Vikøren, T., A. Lillehaug, J. Åkerstedt, T. Bretten, M. Haugum, and M. Tryland. 2008. A severe outbreak of contagious ecthyma (orf) in a free-ranging musk ox (*Ovibos moschatus*) population in Norway. *Veterinary microbiology* 127(1): 10-20.

Vikøren, T., S. Klevar, H. Li, and A.G. Hauge. 2013. Malignant catarrhal fever virus identified in free-ranging musk ox (*Ovibos moschatus*) in Norway. *Journal of Wildlife Disease* 49(2): 447-450.

Webb, S.D., and A.D. Barnosky. 1989. Faunal dynamics of Pleistocene mammals. *Annual Review of Earth and Planetary Sciences* 17: 413-438.

White, R.G., J.E. Rowell, and W.E. Hauer. 1997. The role of nutrition, body condition and lactation on calving success in muskoxen. *Journal of Zoology* 243: 13-20.

Wilkinson, P.F. 1971. The first verified occurrence of twinning in the muskox. *Journal of Mammalogy* 52: 238.

Wilson, K. 1992. Spatial scales of muskox resource selection in late winter. MS Thesis. Fairbanks Alaska USA: University of Alaska Fairbanks.

Ytrehus, B., T. Bretten, B. Bergsjø, and K. Isaksen. 2008. Fatal pneumonia epizootic in musk ox (*Ovibos moschatus*) in a period of extraordinary weather conditions. *Ecohealth* 5(2): 213-223.

Zarnke, R.L., R.A. Dieterich, K.A. Neiland, and G. Ranglak. 1983. Serologic and experimental investigations of contagious ecthyma in Alaska. *Journal of Wildlife Disease* 19: 170-174.

Zarnke, R.L., H. Li, and T.B. Crawford. 2002. Serum antibody prevalence of malignant catarrhal fever viruses in seven wildlife species from Alaska. *Journal of Wildlife Disease* 38(3): 500-504.

REFERENCES cited in supplementary materials Excel Table S3

Aastrup, P., Nielsen, A.B., and Nygaard, K.H. 1988. Miljøundersøgelser ved Safartoq 1986-87. Vildt og vegetation. *Grønlands Miljøundersøgelser.* 54 pp. (in Danish).

Anderson, M. 2014. Distribution and abundance of Peary caribou (*Rangifer tarandus pearyi*) and muskoxen (*Ovibos moschatus*) on the Bathurst Island Group, May 2013. Nunavut Department of Environment, Wildlife Research Section, Status Report, Igloolik, Canada.

Anderson, M. 2016a. Distribution and abundance of Peary caribou (*Rangifer tarandus pearyi*) on Lougheed Island, July 2016. Nunavut Department of Environment, Wildlife Research Section, Status Report 2016-02, Igloolik, Canada.

Anderson, M. 2016b. Distribution and abundance of Peary caribou (*Rangifer tarandus pearyi*) and muskoxen (*Ovibos moschatus*) on Devon Island, March 2016. Nunavut Department of Environment, Wildlife Research Section, Status Report 2016-01, Igloolik, Canada.

Anderson, M. 2016c. Distribution and abundance of muskoxen (*Ovibos moschatus*) and Peary caribou (*Rangifer tarandus pearyi*) on Prince of Wales, Somerset, and Russell islands, August 2016. Nunavut Department of Environment, Wildlife Research Section, Status Report 2016-06, Igloolik, Canada.

Anderson, M., and M.C.S. Kingsley. 2015. Distribution and abundance of Peary caribou (*Rangifer tarandus pearyi*) and muskoxen (*Ovibos moschatus*) on southern Ellesmere Island, March 2015. Nunavut Department of Environment, Wildlife Research Section, Status Report, Igloolik, Canada.
Anderson, R.M. 1930. Notes on the muskox and caribou. In: *Conserving Canada's muskoxen, being an account of an investigation of Thelon Game Sanctuary 1928-29*, ed. W.H.B. Hoar, 41-48. Ottawa, Canada.
Bradley, M., A. Gunn, and J Dragon. 2001. Numbers and abundance of muskoxen, east of Artillery Lake, NWT, July 1998. Manuscript report No. 141, Government of the Northwest Territories, Yellowknife, NT. Canada.
Campbell, M., and M. Setterington. 2001. The re-evaluation of Kivalliq and northeast Ktikmeot muskox (*Ovibos moschatus*) population, management zones, and quotas. Technical Report Series 2001. No. 1-02. 97 pp.
Case, R. 1992. Distribution and abundance of muskoxen on Devon Island, NWT, August 1990. Department of Renewable Resources, Government of the Northwest Territories, Manuscript Report 58, Yellowknife, Canada.
Case, R., and T. Ellsworth. 1991. Distribution and abundance of muskoxen and Peary caribou on southern Ellesmere Island, NWT, July 1989. Department of Renewable Resources, Government of the Northwest Territories, Manuscript Report 41, Yellowknife, Canada.
Case, R.L., and R. Graf. 1986. Abundance and distribution of muskoxen in central Keewatin, NWT. DRWED, Yellowknife, Northwest Territories Government. NWT File Report No. 63. 19pp.
Case, R.L., and K.G. Poole. 1985. Distribution, abundance and composition of muskoxen north of Great Bear Lake, March 1983. Department of Renewable Resources, Government of the Northwest Territories, File Report No.51. 48 pp.
Cuyler, C. 2012. Caribou & muskoxen harvest advice for autumn 2012 / winter 2013. Advisory document for the Greenland Government. 20 April 2012. Pinngortitaleriffik – Greenland Institute of Natural Resources, Nuuk. 9 pp.
Cuyler, C., M. Rosing, H. Mølgaard, R. Heinrich, J. Egede, and L. Mathæussen. 2009. Incidental observations of muskox, fox, hare, ptarmigan and eagle during caribou surveys in West Greenland. Greenland Insitute of Natural Resources. Technical Report No. 75.
Cuyler, C., K.S. Virk, T.B. Clausen, and F.H. Jensen. 2016. 2015 Status Cape Atholl muskoxen (*Ovibos moschatus*) Thule region Greenland. Greenland Institute of Natural Resources. Technical Report No. 96.
Davison, T., and M. Branigan. 2014. Aerial Survey of Muskoxen (Ovibos moschatus) in the Mainland of the Inuvialuit Settlement Region, March 2009. Government of the Northwest Territories. Manuscript Report No. 243 pp.11
Davison, T., J. Pongracz, and J. Williams. 2013. Population survey of Peary caribou (Rangifer tarandus pearyi) and muskoxen (Ovibos moschatus) on Banks Island, Northwest Territories, July 2010. Rangifer 33, Special Issue No.21: 135-140.
Davison, T., and J. Williams. 2013. Peary caribou (*Rangifer tarandus pearyi*) and muskoxen (*Ovibos moschatus*) on northwest Victoria Island, Northwest Territories. *Rangifer* 33, Special Issue No.21: 129-134.
Davison, T., and J. Williams. 2016. Peary Caribou and Muskox Survey of the Melville-Prince Patrick Complex, Northwest Territories and Nunavut, Summer 2012. Department of Environment and Natural Resources, Government of the Northwest Territories. Manuscript Report No. 259: 26 pp.
Davison, T., J. Williams, and J. Adamczewski. 2017. Aerial Survey of Peary Caribou (Rangifer tarandus pearyi) and Muskoxen (Ovibos moschatus) on Banks Island, July 2014. Department of Environment and Natural Resources, Government of the Northwest Territories. Manuscript Report No 270: 25 pp.

Dumond, M. 2006. Muskoxen abundance and distribution, and caribou distribution and calving areas on Boothia Peninsula, Nunavut. Status Report 20. Nunavut Department of Environment, Kugluktuk, NU. 6 pp.

Dumond, M. 2007. Muskox distribution and abundance in the area west of the Coppermine river, Kitikmeot region, Nunavut (May 2007). Department of Environment, Government of Nunavut, Kugluktuk, Nunavut Canada. 29 pp.

Ferguson, M.A.D. 1987. Status of Peary caribou and muskox populations on Bathurst Island, N.W.T., August 1981. *Arctic* 40: 131-137.

Fischer, C.A., and E.A. Duncan. 1976. Ecological studies of caribou and muskoxen in the Arctic Archipelago and northern Keewatin. Renewable Resources Consulting Services Ltd, Canada. 194 pp.

Ford, N., M. Campbell, and D. Lee. 2012. A Final Report to the Nunavut Wildlife Management Board on the Re- evaluation of Muskoxen (*Ovibos moschatus*) Populations in the Kivalliq Region. Report # 2-10-08, January 2012. Prepared by: The Rankin Inlet HTO, Nunavut Department of Environment Kivalliq Region, & Nunavut Tunngavik Inc. Rankin Inlet, NU.

Fournier, B., and A. Gunn. 1998. Muskox numbers and distribution in the Northwest Territories, 1997. Northwest Territories Department of Resources, Wildlife and Economic Development. File Rep. No. 121. 55 pp.

Fraser, P., A. Gunn, and B. McLean. 1992. Abundance and distribution of Peary Caribou and Muskoxen on Banks Island, NWT, June 1991. Renewable Resources, Government of the Northwest Territories. Manuscript Report No. 63. 18pp.

Freeman, M.M.R. 1971. Population characteristics of musk-oxen in the Jones Sound region of the Northwest Territories. *Journal of Wildlife Management* 35: 103-108.

Gorn, T. 2011. Unit 22 Muskox. Pages 16-47. In P. Harper, editor. Muskox management report of survey and inventory activities, 1 July 2008 - 30 June 2010. Alaska Department of Fish and Game, Project 16.0, Juneau, Alaska, USA.

Gorn, T., and W.R. Dunker. 2015. Unit 22 muskox. Chapter 2, pages 1-44 in P. Harper and L/A. McCarthy, editors. Muskox management report of survey and inventory activities 1 July 2012-30 June 2014. Alaska Department of Fish and Game, Species Management Report ADF&G/DWC/SMR-2015-2, Juneau, Alaska, USA.

Graf, R., and C. Shank. 1989. Abundance and distribution of muskoxen near Artillery Lake, NWT, March 1989. Northwest Territories Department of Renewable Resources File Report No. 80. 19pp.

Gray, D.R. 1972. Winter research on the muskox on Bathurst Island, 1970-71. Canadian Wildlife Service unpublished report, Edmonton, Canada.

Gunn, A. 1995. Distribution and abundance of muskoxen west of Coppermine, N.W.T. 1987-88. File Report No109. Department of Resources, Wildlife and Economic Development, Government of the Northwest Territories. Yellowknife. 28pp.

Gunn, A. 2007. Distribution and abundance of muskoxen on northwestern Victoria Island, Nunavut, 1989. Department of Environment and Natural Resources, Government of the Northwest Territories, Yellowknife, NT. Manuscript Report unpublished.

Gunn, A., and R. Case. 1984. Numbers and distribution of muskoxen in the Queen Maud Gulf area, July 1982. Northwest Territories Department of Renewable Resources File Report No. 39. 56 pp.

Gunn, A., and R. Decker. 1984. Numbers and distributions of Peary caribou and muskoxen in July 1980 on Prince of Wales, Russell, and Somerset Islands, N.W.T. Northwest Territories Department of Renewable Resources, File Report 38, Yellowknife, Canada. 56 pp.

Gunn, A. and J. Dragon. 1998. Status of caribou and muskox populations within the Prince of Wales Island – Somerset Island – Boothia Peninsula Complex, NWT, July-August 1995. Northwest Territories Department of Resources, Wildlife and Economic Development, File Report 122, Yellowknife, Canada. 45 pp.

Gunn, A. and J. Dragon. 2002. Peary caribou and muskox abundance and distribution on the Western Queen Elizabeth Islands, Northwest Territories and Nunavut (June-July 1997). Northwest Territories Department of Resources, Wildlife and Economic Development File Report 130, Yellowknife, Canada.

Gunn, A., J. Eamer, P. Reynolds, T.P. Sipko, and A.R. Gruzdev. 2013. Muskoxen. In: Arctic Report Card 2013. http:77www.arctic.noaa.gov/reportcard.

Gunn, A., K. Lambert, and R. Morrison. 1996. Distribution and abundance of muskoxen on Adelaide Peninsula, NWT, 1986 and 1992. Northwest Territories Department of Renewable Resources File Report No. 117. 20 pp.

Gunn, A., and J. Lee. 2000. Distribution and abundance of muskoxen, on northeast Victoria Island, N.W.T. Northwest Territories Department of Resources, Wildlife and Economic Development. Manuscript Rep. No. 119. 12pp.

Gunn, A., and B. R. Patterson. 2012. Distribution and abundance of muskoxen on southeastern Victoria Island, Nunavut, 1988 – 1999. Department of Environment and Natural Resources, Government of the Northwest Territories, Yellowknife, NT. Manuscript Report No. 222. 41pp.

Gunn, A., J. Williams, and R. Shank. 2009. Distribution and abundance of muskoxen in the Beaverhill Lake area (2000) and Thelon Wildlife Sanctuary (1994), Northwest Territories. Department of Environment and Natural Resources, Government of the Northwest Territories, Yellowknife, Canada. Manuscript Report No. 190.

Hubert, B. 1975. Caribou and muskox survey, Prince of Wales and Somerset Islands. Northwest Territories Wildlife Service unpublished data and maps, Yellowknife, Canada.

Hubert, B. A. 1977. Estimated productivity of muskox on Truelove Lowland. In: *Truelove Lowland, Devon Island, Canada: a High Arctic ecosystem*, ed. L.C. Bliss, 467-491. Edmonton, Canada: University of Alberta Press.

Hughes, L.J. 2016. Units 23 and 26A muskox. Chapter 3, Pages 1-19 *In* P. Harper and L/A. McCarthy, editors. Muskox management report of survey and inventory activities 1 July 2012-30 June 2014. Alaska Department of Fish and Game, Species Management Report ADF&G/DWC/SMR-2015-2, Juneau

Jenkins, D., M. Campbell, G. Hope, J. Goorts, and P. McLoughlin. 2011. Recent trends in abundance of Peary Caribou (*Rangifer tarandus pearyi*) and muskoxen (*Ovibos moschatus*) in the Canadian Arctic Archipelago, Nunavut. Department of Environment, Government of Nunavut, Wildlife Report 1, Pond Inlet, Canada.

Jingfors, K. 1984. Abundance, composition and distribution of muskoxen on Southeastern Victoria Island. N.W.T. Wildlife Service File Report No. 36. 245 pp.

Jingfors, K. 1985. Abundance and distribution of muskoxen on northwestern Victoria Island. Department of Renewable Resources, File Report 47, 22pp.

Jones, P., and P. Perry. 2011. Unit 18 Muskox. Pages 1-11, in P. Harper, editor. Muskox management report of survey and inventory activities, 1 July 2008 - 30 June 2010. Alaska Department of Fish and Game, Project 16.0, Juneau, Alaska, USA.

Jones, P., P. Reynolds, T. Gorn, J. Dau, P. Perry, B. Lenart, B. Dunker, G. Carroll, M. Suitor, E. Wald, C. Westing, B. Saito, and I. Hughes. 2016. Status of Muskox in Alaska. Poster. International Muskox Health Ecology Symposium. 7-10 November 2016. Calgary, Alberta, Canada.

Landa, A., S.R. Jeremiassen, and R. Anderson. 2000. Rensdyr of moskusokser I Inglefield Land, Nordvestgrønland. Teknisk rapport nr. 31. Pinngortitaleriffik, Grønlands Naturinstitut, Nuuk, Greeland. (in Danish).

Larter, N.C. 1999. An aerial survey of muskoxen in the Paulatuk area of the Inuvialuit Settlement Region, March 1997. Department of Resources, Wildlife, and Economic Development, Government of the Northwest Territories, Inuvik, NWT. Manuscript Report No. 116. 27pp

Latour, P.B. 1985. Population estimates for Peary caribou and muskoxen on Banks Island in 1982. NWT Wildlife Services, Government of the Northwest Territories. File Report No. 49. 21pp. Reanalyzed in Nagy J. A., P. Latour and W. H. Wright. 2009. Population Estimates for Peary Caribou and Muskox

Leclerc, L.-M. 2015a. Muskox (*Ovibos moschatus*) distribution and abundance, muskox management units MX-10 subdivision, King William Island, September 2013. Status Report 2016. Nunavut Department of Environment, Wildlife Research Section, Kugluktuk, Nunavut. Canada.

Leclerc, L.-M. 2015b. Muskox (*Ovibos moschatus*) distribution and abundance, muskox management units MX-11 subdivision, Kugluktuk, September 2013. Status Report 2016. Nunavut Department of Environment, Wildlife Research Section, Kugluktuk, Nunavut. Canada.

Leclerc, L.-M. 2015c. Muskox (*Ovibos moschatus*) distribution and abundance, muskox management units MX-07, Victoria Island, September 2013-2014. Status Report 2016. Nunavut Department of Environment, Wildlife Research Section, Kugluktuk, Nunavut. Canada.

Lenart, E.A. 2015. Units 26B and 26C muskox. Chapter 4, pages 1-26 *In* P. Harper and L.A. McCarthy, editors. Muskox management report of survey and inventory activities 1 July 2012-30 June 2014. Alaska Department of Fish and Game, Species Management Report ADF&G/DWC/SMR-2015-2, Juneau

Marques, T.A. 2018. Estimating caribou abundance for GINR's 2018 West Greenland caribou survey. CREEM Report 2018-3. Report produced for GINR research contract between CREEM and GINR.

McLean, B.D. 1992. An aerial survey of muskoxen north of Great Bear Lake, August 1987. Renewable Resources, Government of the Northwest Territories. File Report No. 103. 19pp.

Mclean, B., K. Jingfors, and R. Case. 1986. Abundance and Distribution of Muskoxen and Caribou on Banks Island, July 1982. Renewable resources, Government of the Northwest Territories. File Report No. 64. 45pp.

Mclean, B.D., and F. Fraser. 1992. Abundance and distribution of Peary Caribou and muskoxen on Banks Island, NWT, June 1989. Renewable resources, Government of the Northwest Territories. File Report No. 106. 28pp.

Miller, F.L. 1987a. Peary caribou and muskoxen on Bathurst, Alexander, Marc, Massey, Vanier, Cameron, Helena, Lougheed, and Edmund Walker Islands, Northwest Territories, July 1985. Canadian Wildlife Service, Prairie and Northern Region Technical Report Series 20, Edmonton, Canada.

Miller, F.L. 1987b. Peary caribou and muskoxen on Prince Patrick Island, Eglinton Island, and Emerald Isle, Northwest Territories, July 1986. Technical Report Series No. 29. Canadian Wildlife Service, Prairie & Northern Region, Edmonton, Alberta. 65 pp.

Miller, F.L. 1988. Peary caribou and muskoxen on Melville and Byam Martin islands, Northwest Territories, July 1987. Technical Report Series No. 37. Canadian Wildlife Service, Prairie & Northern Region, Edmonton, Alberta. 58 pp.

Miller, F.L. 1989. Re-evaluation of the status of Peary caribou and muskox populations within the Bathurst Island complex, Northwest Territories, July 1988. Canadian Wildlife Service, Prairie and Northern Range Technical Report 78, Edmonton, Canada.

Miller, F.L. 1995. Peary caribou conservation studies, Bathurst Island complex, Northwest Territories, July-August 1993. Canadian Wildlife Service, Prairie & Northern region Technical Report Series 230, Edmonton, Canada.

Miller, F.L. 1997. Peary caribou conservation studies, Bathurst Island Complex, Northwest Territories, April-August 1994 and June-July 1995. Canadian Wildlife Service, Prairie and Northern Region Technical Report Series 295, Edmonton, Canada.

Miller, F.L. 1998. Status of Peary caribou and muskox populations within the Bathurst Island complex, south-central Queen Elizabeth Islands, Northwest Territories, July 1996. Technical Report Series 317, Canadian Wildlife Service, Prairie and Northern Region, Edmonton, Canada.

Miller, F.L. and R.H. Russell. 1976. Distributions, movements, and numbers of Peary caribou and muskoxen on the western Queen Elizabeth Islands, Northwest Territories, 1972-1974. Canadian Wildlife Service, completion report, Edmonton, Canada.

Miller, F.L., R.H. Russell and A. Gunn. 1977. Distribution, movements, and numbers of Peary caribou and muskoxen on Western Queen Elizabeth Islands, NWT 1972-74. Canadian Wildlife Service Report Series 40, Edmonton, Canada.

Nagy, J.A., C. Bucher, and W.H. Wright. 2013. An aerial survey for muskoxen in the Inuvialuit Settlement Region and Tuktut Nogait National Park, 2002. Environment and Natural Resources, Government of the Northwest, Territories. Manuscript Report 226. 36pp.

Nagy, J.A., A. Gunn, and W.H. Wright. 2009a. Population estimates for Peary caribou and muskox on Banks Island, NWT, August 1992. Environment and Natural Resources, Government of the Northwest Territories. Manuscript Report No. 198. 41pp.

Nagy, J.A., A. Gunn, and W.H. Wright. 2009b. Population estimates for Peary caribou and muskox on Banks Island, NWT, July 2005. Environment and Natural Resources, Government of the Northwest Territories. Manuscript Report No. 200. 47pp.

Nagy, J.A., A. Gunn, and W.H. Wright. 2009c. Population Estimates for Peary Caribou (Minto Inlet Herd), Dolphin and Union Caribou, and Muskox on Northwest Victoria Island, NT, July 2005. Department of Environment and Natural Resources, Government of the Northwest Territories, Inuvik and Yellowknife. Manuscript Report 203. 39pp.

Nagy, J.A., N. Larter, and W.H. Wright. 2006. Population estimates for Peary caribou and muskox on Banks Island, NT, July 2001. Environment and Natural Resources, Government of the Northwest Territories. Manuscript Report No. 199. 47pp.

Nagy, J.A., N. Larter, and W.H. Wright. 2009a. Population Estimates for Peary Caribou (Minto Inlet Herd), Dolphin and Union Caribou, and Muskox on Northwest Victoria Island, NT, July 1998. Department of Environment and Natural Resources, Government of the Northwest Territories, Inuvik and Fort Simpson. Manuscript Report 201. 29pp.

Nagy, J.A., N. Larter, and W.H. Wright. 2009b. Population Estimates for Peary Caribou (Minto Inlet Herd), Dolphin and Union Caribou, and Muskox on Northwest Victoria Island, NT, July 2001. Department of Environment and Natural Resources, Government of the Northwest Territories, Inuvik and Yellowknife. Manuscript Report 202. 36pp.

Nagy, J.A., N. Larter, and W.H. Wright. 2013a. Population estimates for Peary caribou and muskox on Banks Island, NWT, July 1994. Environment and Natural Resources, Government of the Northwest Territories. Manuscript Report No. 223. 50pp.

Nagy, J.A., N. Larter, and W.H. Wright. 2013b. Population estimates for Peary caribou and muskox on Banks Island, NWT, July 1998. Environment and Natural Resources, Government of the Northwest Territories. Manuscript Report No. 224. 48pp.

Nagy, J.A., P. Latour, and W.H. Wright. 2009. Population estimates for Peary caribou and muskox on Banks Island, NT, July 1982 – A Retrospective Analysis. Environment and Natural Resources, Government of the Northwest Territories. Manuscript Report No. 197. 52pp

Olesen, C.R. 1993. Rapid population increase in an introduced muskox population, West Greenland. *Rangifer* 13(1): 27-32.

Pattie, D.L. 1990. Muskox (*Ovibos moschatus* Zimmermann) populations on the northern east coast of Devon Island. In: *Canada's missing dimension, science and history in the Canadian arctic islands, Volume II*, ed. C.R. Harrington, 633-641. Ottawa, Canada: Canadian Museum of Natural Science.

Riewe, R.R. 1973. Final report on a survey of ungulate populations on the Bjorne Peninsula, Ellesmere Island: determination of numbers and distribution and assessment of the effects of seismic activities on the behaviour of these populations. University of Manitoba, Report fulfilling contract YK-73/74-020, Winnipeg, Canada.

Shank, C., and R. Graf. 1992. Abundance and distribution of muskoxen near Aylmer Lake, NWT, July 1991. Northwest Territories Department of Renewable Resources Manuscript Report No. 56. 16pp.

Slaney, F.F. and Co. 1975a. Peary caribou and muskoxen and Panarctic's seismic operations on Bathurst Island, N.W.T., 1974. Report submitted to Panarctic Oils Ltd., Calgary, Canada.

Slaney, F.F. and Co. 1975b. Muskoxen calving survey, Bathurst Island, N.W.T, April-May 1975. Report submitted to Panarctic Oils Ltd., Calgary, Canada.

Spencer, D.L., and C.J. Lensink. 1970. The muskox of Nunivak Island, Alaska. *Journal of Wildlife Management* 34(1): 1-15.

Tener, J.S. 1963. Queen Elizabeth Islands game survey, 1961. Canadian Wildlife Service Occasional Paper 4, Edmonton, Canada.

Thing, H., P. Henrichsen, and P. Lassen. 1984. Status of the muskox in Greenland. *Biol. Pap. Univ. Alaska Spec. Rep.* No 4:1-6

Thulin, C.G., L. Englund, G. Ericsson, and G. Spong. 2011. The impact of founder events and introductions on genetic variation in the muskox *Ovibos moschatus* in Sweden. *Acta Theriologica* 56: 305-314.

Urquhart, D.R. 1982. Life history and current status of muskox in the NWT. Northwest Territories Department of Renewable Resources Report, Yellowknife, Canada.

Veitch, A.M. 1997. An aerial survey for muskoxen in the northern Sahtu Settlement Area, March 1997. Department of Resources, Wildlife, and Economic Development, Government of the Northwest Territories, Norman Wells, NWT. Manuscript Report No. 103. 42 pp.

Vibe, C. 1986. Tilbage til de gamle græsgange. *Augut*. Pages 6-7. (in Danish).

Vibe, C. 1971. Moskusoksen. –In: A. Nørrevang, T.J. Meyer, and S. Christensen (eds.). *Danmarks natur, Grønland og Færoerne*. Politikens Forlag. Pages 401-404. (in Danish).
